# Bioorthogonal Uncaging of the Active Metabolite of Irinotecan by Palladium‐Functionalized Microdevices

**DOI:** 10.1002/chem.201803725

**Published:** 2018-11-08

**Authors:** Catherine Adam, Ana M. Pérez‐López, Lloyd Hamilton, Belén Rubio‐Ruiz, Thomas L. Bray, Dirk Sieger, Paul M. Brennan, Asier Unciti‐Broceta

**Affiliations:** ^1^ Cancer Research UK Edinburgh Centre, MRC Institute of Genetics and Molecular Medicine University of Edinburgh UK; ^2^ Centre for Neurogeneration, The Chancellor's Building University of Edinburgh UK; ^3^ Centre for Clinical Brain Sciences University of Edinburgh UK

**Keywords:** anticancer drugs, bioorthogonal catalysis, combination therapy, irinotecan, palladium

## Abstract

SN‐38, the active metabolite of irinotecan, is released upon liver hydrolysis to mediate potent antitumor activity. Systemic exposure to SN‐38, however, also leads to serious side effects. To reduce systemic toxicity by controlling where and when SN‐38 is generated, a new prodrug was specifically designed to be metabolically stable and undergo rapid palladium‐mediated activation. Blocking the phenolic OH of SN‐38 with a 2,6‐bis(propargyloxy)benzyl group led to significant reduction of cytotoxic activity (up to 44‐fold). Anticancer properties were swiftly restored in the presence of heterogeneous palladium (Pd) catalysts to kill colorectal cancer and glioma cells, proving the efficacy of this novel masking strategy for aromatic hydroxyls. Combination with a Pd‐activated 5FU prodrug augmented the antiproliferative potency of the treatment, while displaying no activity in the absence of the Pd source, which illustrates the benefit of achieving controlled release of multiple approved therapeutics—sequentially or simultaneously—by the same bioorthogonal catalyst to increase anticancer activity.

## Introduction

Bioorthogonal reactions are designed to take place in cells and organisms without interfering with biological functions.^1^ In the pursuit of exploiting such processes in cancer therapy, reactions and tools that were once exclusively used to synthesize drugs in chemistry labs have been recently adapted to perform such tasks in living systems.^2^, ^3^, ^4^, ^5^, ^6^, ^7^, ^8^, ^9^, ^10^, ^11^, ^12^, ^13^, ^14^, ^15^, ^16^, ^17^, ^18^, ^19^, ^20^ Chemotherapeutics as doxorubicin,^2^, ^3^, ^4^, ^5^, ^6^, ^7^ 5FU,^8^, ^9^, ^10^, ^11^ gemcitabine,^17^ floxuridine,^18^ vorinostat^19^ or nitric oxide precursors^20^ can be “manufactured” from inactive precursors in biological environments through a variety of bio‐independent processes, including click‐to‐release reactions and bioorthogonal organometallic catalysis. In combination with a suitable cancer targeting strategy (e.g. antibody‐based tumor targeting,^15^ enhanced permeability and retention effect,^6^, ^11^ intratumoral implantation),^7^ these highly selective reactions can facilitate the spatially controlled release of one or more therapeutic agents to localize drug activity at the tumor site. While such approaches are yet to demonstrate utility in the clinic, they have the potential to reduce systemic side effects and enhance treatment efficacy by generating greater drug levels at the disease site than can be safely achieved by standard chemotherapy.

Palladium (Pd) catalysts are one of the tools of choice currently under investigation to release caged drugs in vitro and in vivo.^7^, ^8^, ^9^, ^10^, ^11^, ^17^, ^18^, ^19^, ^20^ The selection of this metal is based on its high bio‐compatibility, its versatility to adopt different shapes and sizes and its remarkable capabilities to catalyze *N*‐ and *O*‐dealkylation reactions on manifold types of substrates under physiological conditions.^21^, ^22^, ^23^ Our lab is currently investigating the application of heterogeneous Pd catalysts as implantable devices to mediate drug release with spatiotemporal control and in a catalytic fashion, with the goal of improving the safety profile of chemotherapies without having the short, limited life of other local therapy modalities such as drug eluting devices (carmustine wafers)^24^ or brachytherapy.^25^

**Figure 1:**
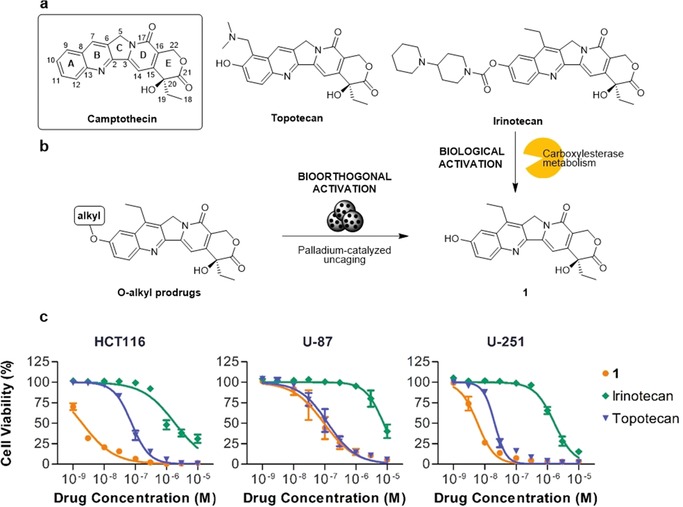
(a) Camptothecin and clinically approved derivatives topotecan and irinotecan. (b) Biological (enzymatic) activation of irinotecan to release the active metabolite **1** and bioorthogonal (palladium‐catalyzed) activation of alkylated prodrugs of **1**. (c) Dose‐response curves for HCT116, U‐87, and U‐251 cells after 5 d treatment with **1**, topotecan and irinotecan. Error bars: ±SEM, *n*=3.

The camptothecin‐derived topoisomerase I inhibitors topotecan and irinotecan (Figure 1 a) are anticancer drugs used in the treatment of ovarian (topotecan), lung (both) and colon (irinotecan) cancers. Irinotecan crosses the blood brain barrier^26^ and displays high cytotoxic activity against glioblastoma cells with multi‐drug resistance to other therapies.^27^ In addition, several Phase II clinical trials have reported that the combination of bevacizumab and irinotecan shows promising activity in recurrent malignant glioma with a modest increment in median survival.^28^, ^29^, ^30^ The side effects of irinotecan treatments are, however, a major concern that limits its therapeutic dose and significantly impacts patient's quality of life. To circumvent this issue, the intratumoral implantation of irinotecan‐loaded drug‐eluting beads has been investigated in patients with recurrent glioblastoma^31^ and colorectal liver metastasis.^32^ While these treatments are well tolerated, the drug is fully cleared from the organism within hours of implantation, thus reducing its anticancer effect.^31^, ^32^ Of note, irinotecan is in fact an orally‐bioavailable prodrug that requires enzymatic conversion into its active metabolite SN‐38 (**1**) to reach its full cytotoxic potential (Figure 1 a).^33^ Although glioma cells have been shown to partly metabolize irinotecan, the drug is primarily metabolized into **1** in the liver, from where it distributes throughout the organism. This further rationalizes the limited effect of irinotecan‐loaded drug‐eluting devices in the treatment of brain cancers.

Building on previous success in the development and bio‐independent release of caged drugs with catalyst‐loaded devices,^7^, ^8^, ^9^, ^10^, ^11^ herein we report the design, synthesis and screening of a novel class of inactive camptothecin derivatives that are selectively converted into cytotoxic **1** by bioorthogonal Palladium chemistry.

## Results and Discussion

### Rationale for prodrug design and synthesis of 2 a–d

Preliminary screening of the anticancer properties of **1**, topotecan and irinotecan against colorectal cancer HCT116 cells, and glioma U‐87 and U‐251 cell lines, confirmed the superior potency of **1** relative to irinotecan (Figure 1 c). Topotecan also has lower potency against the HCT116 cell line and, to a lesser extent, U‐251 when compared to **1** (Figure 1 c), but the activity gap is not as prominent as in irinotecan, which has greater than 80‐fold difference in EC_50_ (Table S1 in the Supporting Information). Encouraged by the dramatic change in activity between compounds that only differ by the chemical group found around the C10 position of the camptothecin scaffold (ring A, Figure 1 a), it was rationalized that the aromatic hydroxyl group of **1** was a convenient handle where bulky masking groups could be incorporated and thereby reduce prodrug‐target interactions.

**Scheme 1:**
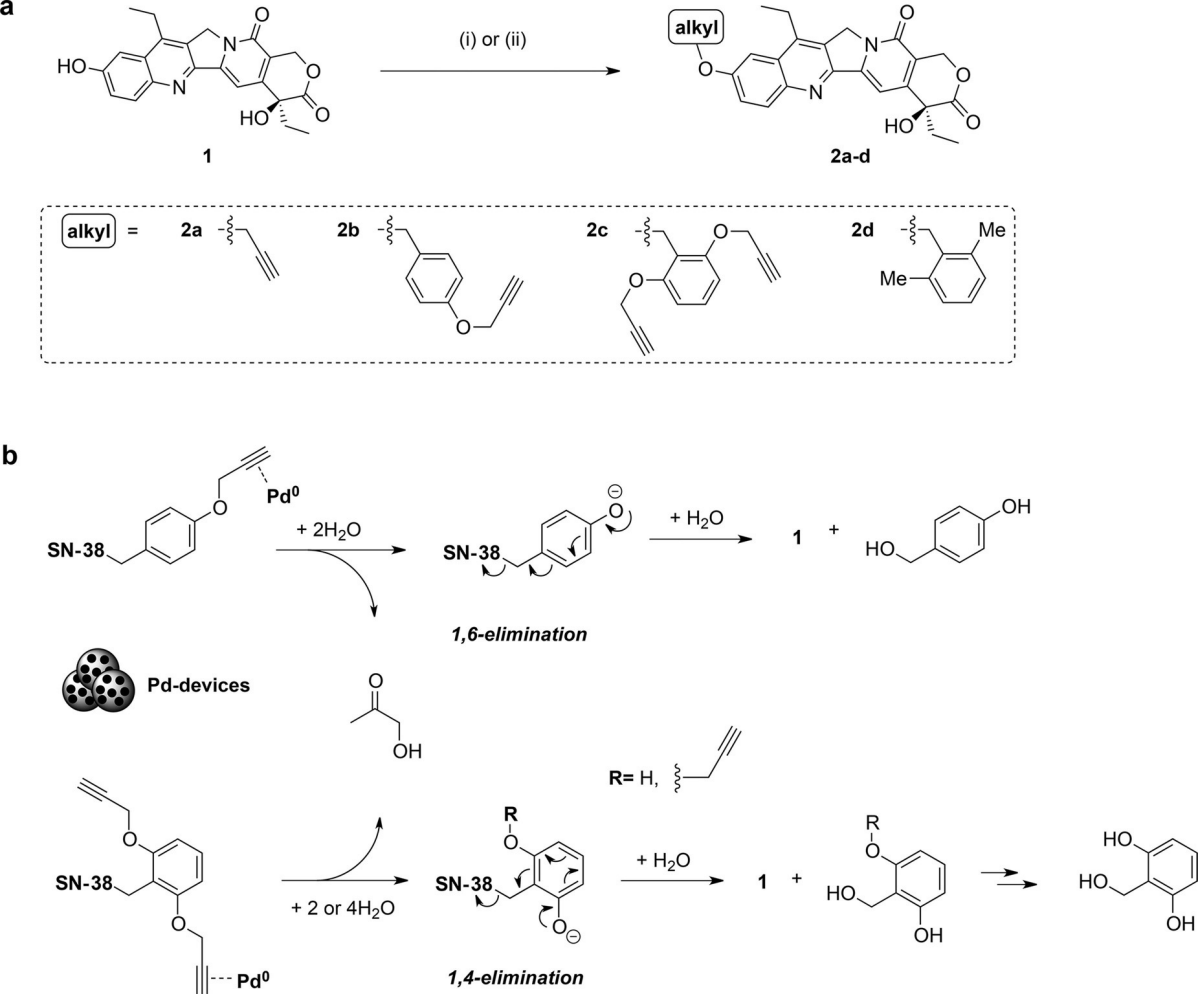
a) Synthesis prodrugs **2 a**–**d**. (i) Alkyl halide, K_2_CO_3_, DMF, rt, overnight; or (ii) alkyl halide, K_2_CO_3_, MeCN, MW, 120 °C, 2 h. b) Proposed Pd‐triggered uncaging mechanism of **2 b**,**c**.

It has been recently reported that the blockade of the NH_2_ group of doxorubicin with an *o*‐(propargyloxy)benzyloxycarbonyl group dramatically reduces the bioactivity of the resulting derivative, while making it activatable by Pd chemistry.^7^ Inspired on this observation, a novel masking group—namely 2,6‐bis(propargyloxy)benzyl—incorporating two symmetrically‐placed Pd‐sensitive triggers was designed to block the OH at C10 of **1** and thereby endow high metabolic stability and increased sensitivity to Pd catalysis. Upon catalyst‐mediated *O*‐dealkylation of either of the propargyl groups, the masking group will eliminate spontaneously to release **1** (see activation mechanism in Scheme 1 b). Derivatives **2 a** and **2 b**, incorporating a propargyl and a 4‐propargyloxybenzyl group, respectively, were used as positive controls. A 2,6‐bis(methoxy)benzyl group was also tested as a steric mimic of the 2,6‐bis(propargyloxy)benzyl group that is unable to be cleaved by Pd chemistry (negative control).

Prodrugs **2 a**–**d** were prepared in a single Williamson ether coupling of **1** and the corresponding alkyl halide in the presence of a base (Scheme 1). Commercially available propargyl bromide and 2,6‐dimethylbenzyl chloride were used in the synthesis of **2 a** and **2 d**, respectively, and benzyl chlorides **5** and **9** (see the Supporting Information) were used for the preparation of **2 b** and **2 c**. Of note, alkylation rates greatly increased under microwave irradiation.

### Cytotoxicity study: 1 vs. 2 a–d

**Figure 2:**
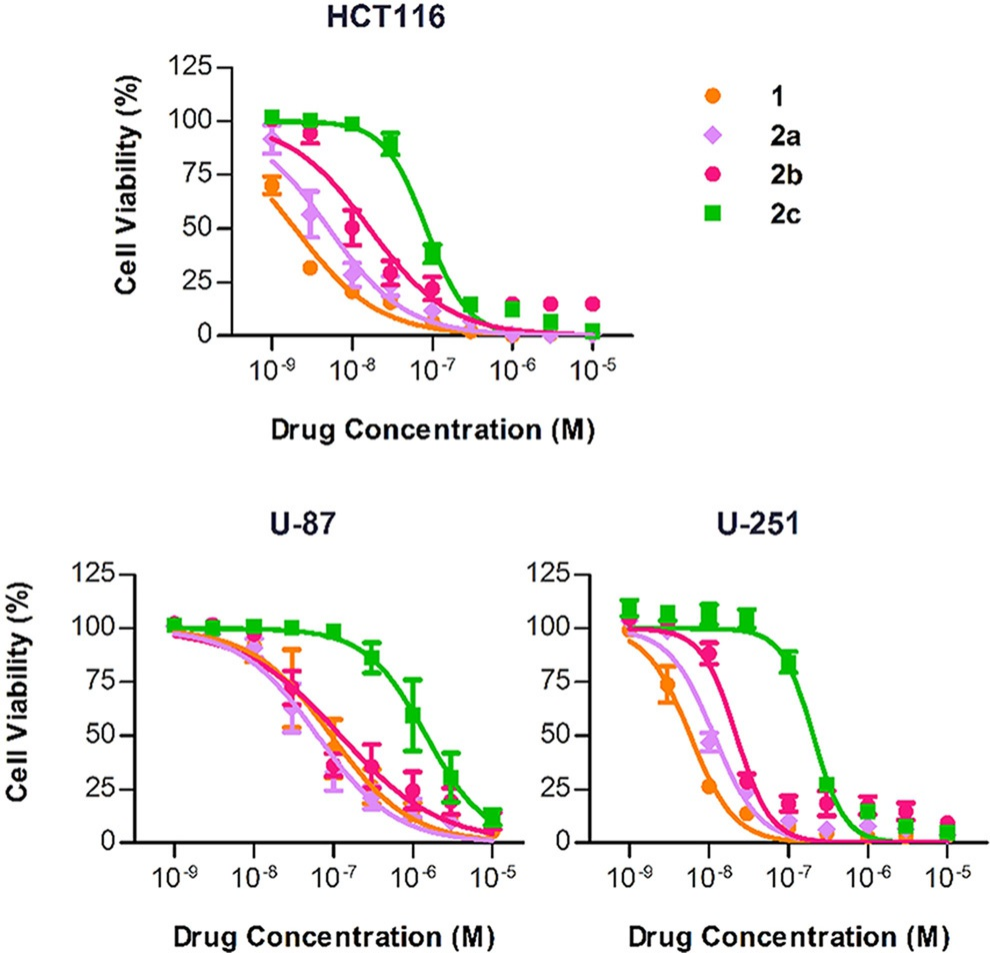
Dose response curves for HCT116, U‐87, and U‐251 cells after 5 d treatment with **1** or **2 a**–**c**. Error bars: ±SEM, *n*=3.

The antiproliferative properties of compounds **2 a**–**d** were tested in cancer cell culture against HCT116, U‐87 and U‐251 cells and compared to **1**. In line with the expectation that steric bulk around the phenol group at C10 reduces compounds’ capacity to interact with its target (topoisomerase I),^34^ it was found that the larger the alkyl group the greater the reduction in cytotoxicity (Figure 2). *O*‐propargylation (**2 a**) of **1** slightly reduced cytotoxicity compared to the parent compound (up to 3‐fold reduction, Table 1). Incorporation of a 4‐propargyloxybenzyl group (**2 b**) had a superior while still minor effect (up to 9‐fold reduction). In contrast, compound **2 c** (containing the novel 2,6‐bis(propargyloxy)benzyl group) led to a much greater reduction in cytotoxicity across the three cell lines (up to 44‐fold reduction) and, thus, was selected for the next phase of investigation. Control compound **2 d** also decreased the experimental cytotoxicity in all cell lines (Figure S**1**) by a similar magnitude to **2 c**. EC_50_ values are shown in Table 1.

**Table 1:** Calculated EC_50_ values [nm] for **1** and **2 a**–**d** in colorectal and glioma cell lines. *n*=3.

Compound	HCT116	U‐87	U‐251
**1**	1.9	94	5.8
**2 a**	5.1	63	12
**2 b**	16	123	22
**2 c**	84	1466	205
**2 d**	104	603	92

### Prodrug activation studies: In vitro and in cell culture

**Figure 3:**
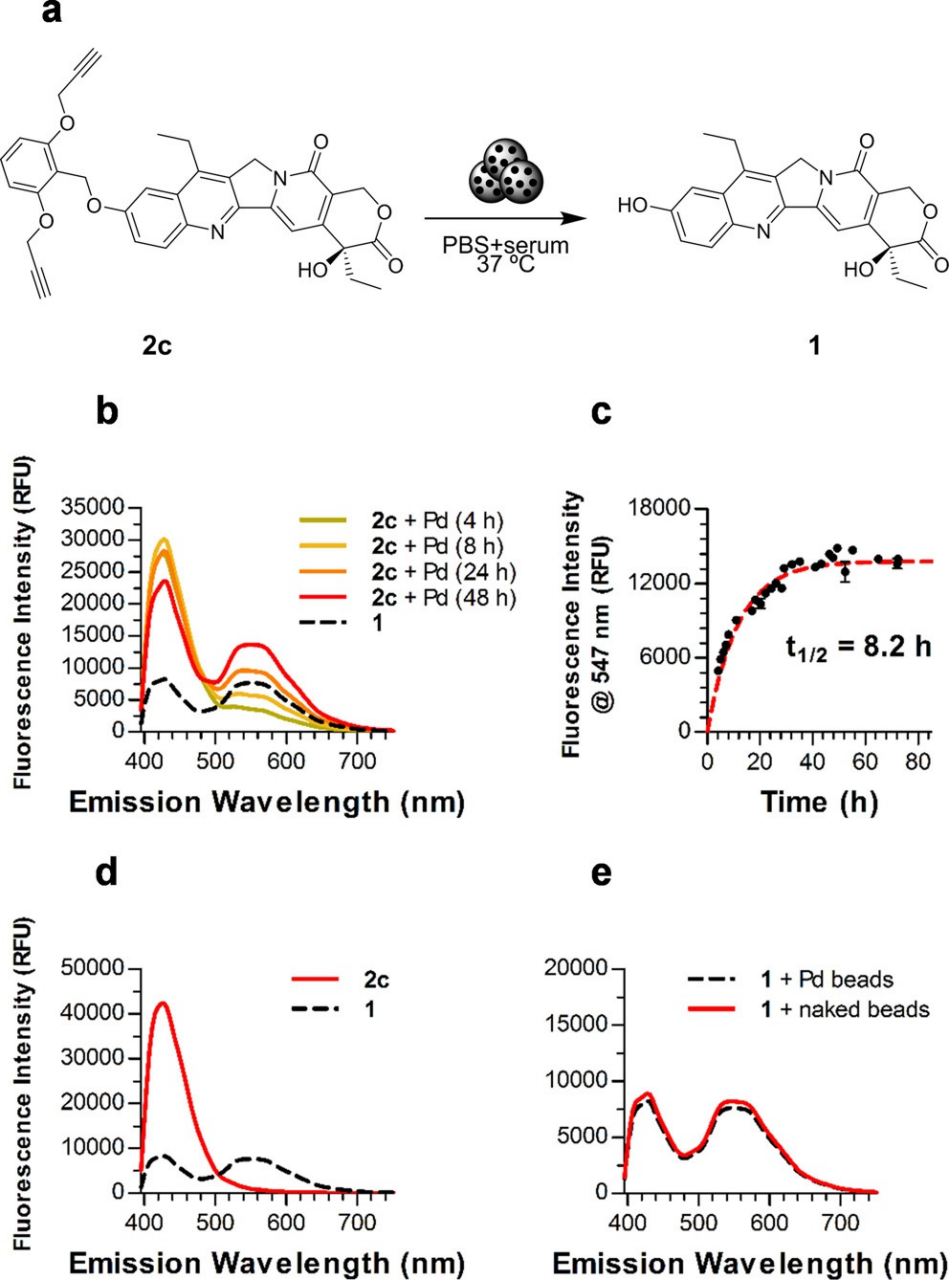
(a) Palladium‐catalyzed conversion of **2 c** into **1**. (**b**,**c**) Rate of transformation of **2 c** (100 μm) into **1** in the presence of Pd^0^ (1 mg mL^−1^ Pd‐devices) in PBS+10 % serum at 37 °C. (b) Monitoring of changes in fluorescence spectra over time. (c) Increase of fluorescence intensity at 547 nm over time (d) Control experiment: fluorescence spectra of **2 c** (100 μm) after 2 d in biocompatible conditions in the presence of naked beads. Black line represents the fluorescent spectra of **1** (100 μm). (e) Control experiment: fluorescence spectra of **1** after 2 d in biocompatible conditions in the presence of Pd‐devices (dotted line) or naked beads (solid line).

Prodrug **2 c** was incubated with 30 μm Tentagel resins loaded with Pd^0^ nanoparticles (Pd‐microdevices, 2 % w/w in Pd)^7^ under physiological conditions to assess its sensitivity to Pd catalysis. Pd‐microdevices were suspended in PBS with 10 % v/v serum containing either **2 c**, **1** (+ve control) or **2 d** (−ve control) and incubated for 2 d at 37 °C. Naked beads (used as purchased prior to Pd loading) were used as a metal‐free control. Reactions were monitored by fluorescence spectroscopy. The characteristic emission spectrum of **1** (*λ*_max_=547 nm, which is absent in **2 c** and **2 d**) facilitated the study of drug release by fluorescence analysis. As shown in Figure 3 b,c, fluorescence signal from uncaged compound **1** rapidly increased over time when **2 c** was incubated with Pd‐microdevices, being patently visible after 4 h. In contrast, incubation of **2 c** alone (Figure S2) or with naked beads (Figure 3 d) under the same conditions did not increase the signal at 547 nm (=no formation of **1**). Similarly, incubation of the control compound **2 d** (Figure S3) with Pd‐resins did not result in the release of **1**. A decrease in the overall fluorescence intensity was observed both in the presence of Pd‐microdevices and naked beads, presumably due to partial compound sequestration by the device. Incubation of unmodified **1** with Pd‐microdevices or naked beads resulted in identical fluorescence spectra (Figures 3 and S4 e), indicating that Pd does not have any effect on the structural integrity of the active drug **1**. The Pd‐mediated conversion of **2 c** into **1** was further confirmed by TLC and HPLC analysis (Figures S5–6).

**Figure 4:**
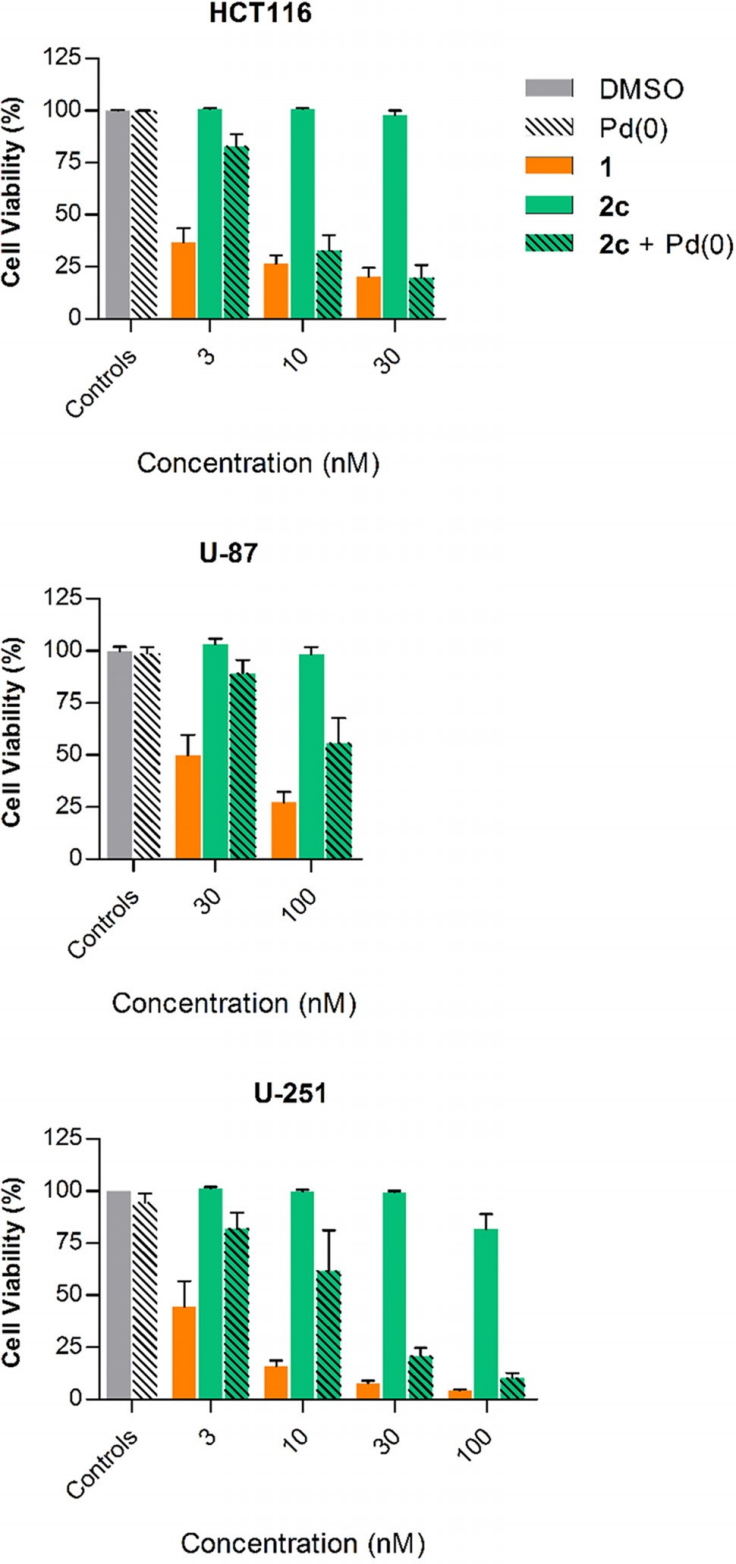
Pd‐catalyzed uncaging of **2 c** in cancer cell culture. Experiments: 0.1 % (v/v) DMSO (control, grey); 1 mg mL^−1^ of Pd‐devices (−ve control, black stripes); **1** (+ve control, orange); **2 c** (−ve control, green); 1 mg mL^−1^ of Pd‐devices +**2 c** (activation assay, green with black stripes). Cell viability was measured at day 5 using PrestoBlue. Error bars: ±SEM, *n*=3.

The bioorthogonal release of cytotoxic **1** from inactive **2 c** by extracellular Pd‐microdevices was then tested in cancer cell culture in HCT116, U‐87 and U‐251 cells (Figure 4). Experiments were conducted under standard cell culture conditions (media supplemented with serum, 5 % CO_2_, 37 °C). Cells were independently treated with Pd‐microdevices (1 mg mL^−1^) or **2 c** (−ve controls) or in combination (activation assay) and compared to treatment with unmodified **1** (+ve control) across a range of concentrations. As expected,^7^, ^8^, ^9^, ^10^, ^11^ the devices (which are larger than cells and remain in the extracellular space) displayed no toxic effect. In contrast, the Pd‐devices/**2 c** combination led to potent antiproliferative effect in all cell lines, slightly lower than that mediated by direct treatment with **1**. Due to the high potency of the active metabolite, nanomolar prodrug concentrations were sufficient to achieve high toxicity, while **2 c** did not display cytotoxicity in the absence of the Pd source. As expected, the antiproliferative properties of **2 d** were not improved in the presence of Pd‐microdevices (see Figure S7).

### Multi prodrug activation study: Co‐treatment with Pro‐5FU

**Figure 5:**
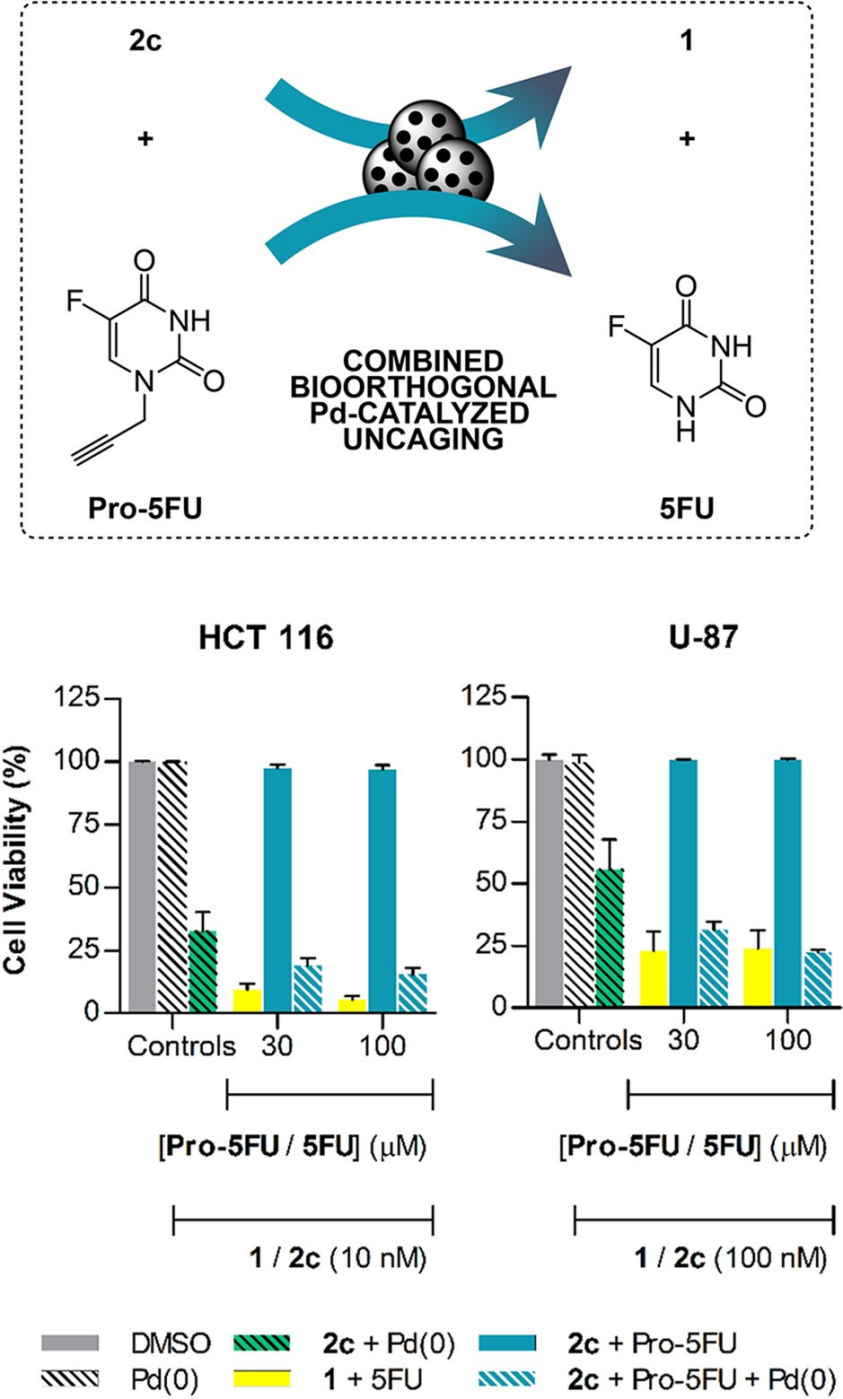
Combined Pd‐catalyzed activation of **2 c** and **Pro‐5FU** in cancer cell culture. Experiments: 0.1 % (v/v) DMSO (control, grey); 1 mg mL^−1^ of Pd‐devices (−ve control, black stripes); 1 mg mL^−1^ of Pd‐devices +**2 c** (+ve control activation, green with black stripes); **1**+**5FU** (+ve control, yellow); **2 c**+**Pro‐5FU** (−ve control, light blue); 1 mg mL^−1^ of Pd‐devices +**2 c**+**Pro‐5FU** (combined activation assay, blue stripes). Cell viability was measured at day 5 using PrestoBlue. Error bars: ± SEM, *n*=3.

Irinotecan is typically administered in combination with 5FU for the treatment of colorectal cancer.^35^ As our lab had previously developed a Pd‐labile prodrug of 5FU, a.k.a. Pro‐5FU (Figure 5),^8^ we were intrigued about the prospect of combining **2 c** and Pro‐5FU in the presence of Pd to simultaneously release two clinically‐used synergistic drugs by the same bioorthogonal triggering mechanism. Such an approach could serve to boost treatment efficacy without compromising safety, an optimal strategy for fighting difficult‐to‐treat cancers. Following the protocol described before, colorectal cancer HCT116 cells were incubated with **2 c** and Pro‐5FU in the presence and absence of Pd‐microdevices. The experiment was also performed in the U‐87 cell line, since the therapeutic effect mediated by the **2 c**/Pd‐devices combination was suboptimal (Figure 4). As shown in Figure 5, no signs of toxicity were observed from the combination of Pro‐5FU (30 or 100 μm) and **2 c** (10 or 100 nm) in either cell line (see Figure S8 for co‐treatment dose‐response curves). Notably, in the presence of the activating device, the combined treatment of the prodrugs elicited superior cytotoxic activity than the **2 c**/Pd‐devices or direct treatment with **1** (Figure 5); evidence that both drugs are concomitantly released by Pd chemistry. Preliminary assessment on the most effective timing of administration of the combination partners against U‐87 cells suggested that compound **2 c** should be administered first (see Figure S9). The potential of manufacturing multiple synergistic drugs at a desired location is one of the most clinically relevant features of this strategy.

## Conclusions

In this manuscript we have reported the first inactive precursor of the potent topoisomerase I inhibitor SN‐38 (**1**) that is specifically released by Pd chemistry. Prodrug **2 c** was successfully masked with a novel alkyl group, 2,6‐bis(propargyloxy)benzyl, which incorporates two Pd‐labile propargyl groups at the *ortho* positions of the benzyl group to increase steric hindrance and augment the rate of cleavage in the presence of Pd catalysts. **2 c** showed low inherent cytotoxicity to colorectal cancer and glioma cells. In the presence of extracellular Pd‐microdevices, the Pd‐labile group at the C10 position of the prodrug was efficiently cleaved to release the cytotoxic compound **1** both in vitro and in cancer cell culture. While previous attempts to make multiple bioactive agents in cell culture have been reported,^36^ herein it is shown for the first time the concomitant uncaging of two drugs used in clinical combinations by the same bioorthogonal method. This study showcases the versatile applicability of using heterogeneous metal catalysts to control the release of therapeutics at desired locations. By intratumoral implantation of the Pd‐devices,^7^ otherwise toxic therapeutic agents such as **1** and 5FU could be generated at the tumor site to mediate focal chemotherapy and thereby reduce systemic side effects.

## Experimental Section

**General**. Reactions requiring anhydrous conditions were carried out under nitrogen in oven‐dried glassware. Dry solvents and reagents were purchased from Acros, Fluorochem, Sigma–Aldrich or VWR and used as received. Irinotecan was purchased from Carbosynth, and topotecan from ABCR. Both were used as purchased. NMR spectra were recorded on a Bruker 500 MHz spectrometer at 300 K and referenced relative to the solvent residual peaks with chemical shifts (*δ*) reported in ppm. Coupling constants (*J*) are reported in Hertz. High resolution mass spectra were measured in a Bruker MicrOTOF II. Analytical and semi‐preparative TLC were performed using Merck TLC Silica Gel 60 F254 plates and visualized by UV light and flash column chromatography using silica gel (220–440 mesh, Sigma–Aldrich). All compounds used in the biological experiments were >95 % pure by UPLC, as measured using a C18 Column, 60 °C; monitoring at 210 nm; eluent A, water with TFA (0.1 %); eluent B, acetonitrile. Method 1 @0.4 mL min^−1^: A/B=95:5 isocratic 0.2 min, 95:5 to 5:95 in 2.3 min, 5:95 isocratic 0.5 min, 5:95 to 95:5 in 0.01 min, 95:5 isocratic 0.49 min. Method 2 @0.2 mL min^−1^: A/B=95:5 isocratic 0.5 min, 95:5 to 5:95 in 10 min, 5:95 isocratic 0.49 min, 5:95 to 95:5 in 0.01 min. Stock solutions (100 mm) were prepared in DMSO diluted to 5 μm in water.

**General method for the synthesis of 2 a**–**d**. Compound **1** (1 equiv) was pre‐stirred with K_2_CO_3_ (1.5 equiv) in DMF or MeCN (20 mL mmol^−1^) before adding the corresponding alkyl halide (1.2 equiv). Reactions were then stirred at ambient temperature overnight or heated under microwave irradiation for 2 h at 120 °C. Solvent was removed under reduced pressure and the crude prodrugs purified by semi‐preparative TLC.

**Synthesis and characterization of 2 c**. 38 mg scale, MeCN, μw, purified 4 % MeOH/CH_2_Cl_2_ to yield a yellow solid (23 mg, 39 %). ^1^H NMR (500 MHz, [D_6_]DMSO) *δ*=8.07 (d, *J=*9.2 Hz, 1 H), 7.68 (d, *J=*2.7 Hz, 1 H), 7.53 (dd, *J=*9.2, 2.7 Hz, 1 H), 7.41 (t, *J=*8.4 Hz, 1 H), 7.29 (s, 1 H), 6.87 (d, *J=*8.4 Hz, 2 H), 6.49 (s, 1 H), 5.44 (d, *J=*1.4 Hz, 2 H), 5.33 (s, 2 H), 5.26 (s, 2 H), 4.88 (d, *J=*2.4 Hz, 4 H), 3.56 (t, *J=*2.4 Hz, 2 H), 3.24 (q, *J=*7.3, 2 H), 1.88 (m, 2 H), 1.33 (t, *J=*7.4 Hz, 3 H), 0.89 ppm (t, *J=*7.3 Hz, 3 H); ^13^C NMR (126 MHz, [D_6_]DMSO) *δ*=173.0, 158.4, 157.6, 156.4, 150.5, 150.0, 146.8, 145.1, 144.5, 131.9, 130.9, 130.3, 128.8, 128.3, 123.1, 118.6, 113.4, 106.8, 105.1, 96.5, 79.6, 78.9, 72.9, 65.7, 56.7, 50.0, 30.7, 22.8, 14.0, 8.2 ppm; HRMS (ESI^+^) *m*/*z* [*M*+H]^+^ calcd for C_35_H_31_N_2_O_7_: 591.2126 found: 591.2149. Purity: 95 % (UPLC, method 2).

## Conflict of interest

The authors declare that compounds **2 b**,**c** are protected under patent application PCT/GB2017/051379.

## Supporting information

As a service to our authors and readers, this journal provides supporting information supplied by the authors. Such materials are peer reviewed and may be re‐organized for online delivery, but are not copy‐edited or typeset. Technical support issues arising from supporting information (other than missing files) should be addressed to the authors.

Supplementary

## References

[1] E. M. Sletten , C. R. Bertozzi , Angew. Chem. Int. Ed. 2009, 48, 6974–6998; 10.1002/anie.200900942 PMC2864149 19714693

[2] R. M. Versteegen , R. Rossin , W. ten Hoeve , H. M. Janssen , M. S. Robillard , Angew. Chem. Int. Ed. 2013, 52, 14112–14116; 10.1002/anie.201305969 24281986

[3] T. Völker , F. Dempwolff , P. L. Graumann , E. Meggers , Angew. Chem. Int. Ed. 2014, 53, 10536–10540; 10.1002/anie.201404547 25138780

[4] S. S. Matikonda , D. L. Orsi , V. Staudacher , I. A. Jenkins , F. Fiedler , J. Chen , A. B. Gamble , Chem. Sci. 2015, 6, 1212–1218. 29560207 10.1039/c4sc02574a PMC5811098

[5] A. M. Pérez-López , B. Rubio-Ruiz , V. Sebastián , L. Hamilton , C. Adam , T. L. Bray , S. Irusta , P. M. Brennan , G. Lloyd-Jones , D. Sieger , J. Santamaría , A. Unciti-Broceta , Angew. Chem. Int. Ed. 2017, 56, 12548–12552; 10.1002/anie.201705609 PMC5655737 28699691

[6] M. A. Miller , B. Askevold , H. Mikula , R. H. Kohler , D. Pirovich , R. Weissleder , Nat. Commun. 2017, 8, 15906. 28699627 10.1038/ncomms15906 PMC5510178

[7] T. L. Bray , M. Salji , A. Brombin , A. M. Pérez-López , B. Rubio-Ruiz , L. C. A. Galbraith , E. E. Patton , H. Y. Leung , A. Unciti-Broceta , Chem. Sci. 2018, 9, 7354–7361. 10.1039/c8sc02291g PMC6237126 30542538

[8] J. T. Weiss , J. C. Dawson , K. G. Macleod , W. Rybski , C. Fraser , C. Torres-Sánchez , E. E. Patton , M. Bradley , N. O. Carragher , A. Unciti-Broceta , Nat. Commun. 2014, 5, 3277. 24522696 10.1038/ncomms4277 PMC3929780

[9] J. T. Weiss , C. Fraser , B. Rubio-Ruiz , S. H. Myers , R. Crispin , J. C. Dawson , V. G. Brunton , E. E. Patton , N. O. Carragher , A. Unciti-Broceta , Front. Chem. 2014, 2, 56. 25121087 10.3389/fchem.2014.00056 PMC4114543

[10] G. Y. Tonga , Y. Jeong , B. Duncan , T. Mizuhara , R. Mout , R. Das , S. T. Kim , Y. C. Yeh , B. Yan , S. Hou , V. M. Rotello , Nat. Chem. 2015, 7, 597–603. 26100809 10.1038/nchem.2284 PMC5697749

[11] M. Hoop , A. S. Ribeiro , D. Rösch , P. Weinand , N. Mendes , F. Mushtaq , X.-Z. Chen , Y. Shen , C. F. Pujante , J. Puigmartí-Luis , J. Paredes , B. J. Nelson , A. P. Pêgo , S. Pané , Adv. Funct. Mater. 2018, 28, 1705920.

[12] M. I. Sánchez , C. Penas , M. E. Vázquez , J. L. Mascareñas , Chem. Sci. 2014, 5, 1901–1907. 24955233 PMC4064253

[13] J. Clavadetscher , S. Hoffmann , A. Lilienkampf , L. Mackay , R. M. Yusop , S. A. Rider , J. J. Mullins , M. Bradley , Angew. Chem. Int. Ed. 2016, 55, 15662–15666; 10.1002/anie.201609837 27860120

[14] B. Li , P. Liu , H. Wu , X. Xie , Z. Chen , F. Zeng , S. Wu , Biomaterials 2017, 138, 57–68. 28554008 10.1016/j.biomaterials.2017.05.036

[15] R. Rossin , R. M. Versteegen , J. Wu , A. Khasanov , H. J. Wessels , E. J. Steenbergen , W. ten Hoeve , H. M. Janssen , A. H. A. M. van Onzen , P. J. Hudson , M. S. Robillard , Nat. Commun. 2018, 9, 1484. 29728559 10.1038/s41467-018-03880-y PMC5935733

[16] S. Alonso-de Castro , A. L. Cortajarena , F. López-Gallego , L. Salassa , Angew. Chem. Int. Ed. 2018, 57, 3143–3147; 10.1002/anie.201800288 PMC5887934 29359850

[17] J. T. Weiss , J. C. Dawson , C. Fraser , W. Rybski , C. Torres-Sánchez , M. Bradley , E. E. Patton , N. O. Carragher , A. Unciti-Broceta , J. Med. Chem. 2014, 57, 5395–5404. 24867590 10.1021/jm500531z PMC4078945

[18] J. T. Weiss , N. O. Carragher , A. Unciti-Broceta , Sci. Rep. 2015, 5, 9329. 25788464 10.1038/srep09329 PMC4365405

[19] B. Rubio-Ruiz , J. T. Weiss , A. Unciti-Broceta , J. Med. Chem. 2016, 59, 9974–9980. 27786474 10.1021/acs.jmedchem.6b01426

[20] T. Lv , J. Wu , F. Kang , T. Wang , B. Wan , J. J. Lu , Y. Zhang , Z. Huang , Org. Lett. 2018, 20, 2164–2167. 29595271 10.1021/acs.orglett.8b00423

[21] J. Li , J. Yu , J. Zhao , J. Wang , S. Zheng , S. Lin , L. Chen , M. Yang , S. Jia , X. Zhang , P. R. Chen , Nat. Chem. 2014, 6, 352–361. 24651204 10.1038/nchem.1887

[22] J. Wang , B. Cheng , J. Li , Z. Zhang , W. Hong , X. Chen , P. R. Chen , Angew. Chem. Int. Ed. 2015, 54, 5364–5368; 10.1002/anie.201409145 25765364

[23] M. Martínez-Calvo , J. R. Couceiro , P. Destito , J. Rodríguez , J. Mosquera , J. L. Mascareñas , ACS Catal. 2018, 8, 6055–6061. 30018848 10.1021/acscatal.8b01606 PMC6038097

[24] J. Perry , A. Chambers , K. Spithoff , N. Laperriere , Curr. Oncol. 2007, 14, 189–194. 17938702 10.3747/co.2007.147 PMC2002480

[25] T. Y. Tam , S. Mukherjee , T. Farrell , D. Morgan , R. Sur , Brachytherapy 2009, 8, 313–317. 19211311 10.1016/j.brachy.2008.12.003

[26] J. J. Vredenburgh , A. Desjardins , D. A. Reardon , H. S. Friedman , Neuro-Oncology 2009, 11, 80–91. 18784279 10.1215/15228517-2008-075 PMC2718962

[27] S. Nakatsu , S. Kondo , Y. Kondo , D. Yin , J. W. Peterson , R. Kaakaji , T. Morimura , H. Kikuchi , J. Takeuchi , G. H. Barnett , Cancer Chemother. Pharmacol. 1997, 39, 417–423. 9054955 10.1007/s002800050592

[28] J. J. Vredenburgh , A. Desjardins , J. E. Herndon , J. M. Dowell , D. A. Reardon , J. A. Quinn , J. N. Rich , S. Sathornsumetee , S. Gururangan , M. Wagner , D. D. Bigner , A. H. Friedman , H. S. Friedman , Clin. Cancer. Res. 2007, 13, 1253–1259. 17317837 10.1158/1078-0432.CCR-06-2309

[29] T. F. Cloughesy , M. D. Prados , P. Y. Wen , T. Mikkelsen , L. E. Abrey , D. Schiff , W. K. Yung , Z. Maoxia , I. Dimery , H. S. Friedman , J. Clin. Oncol. 2008, 26, 2010b.

[30] T. N. Kreisl , L. Kim , K. Moore , P. Duic , C. Royce , I. Stroud , N. Garren , M. Mackey , J. A. Butman , K. Camphausen , J. Park , P. S. Albert , H. A. Fine , J. Clin. Oncol. 2009, 27, 740–745. 19114704 10.1200/JCO.2008.16.3055 PMC2645088

[31] G. Cruickshank, D. Ngoga, A. Detta, A. Lewis, R. Holden, O. Fayaye, Abstracts from the BNOS 2017 Meeting June 21–23, 2017 John McIntyre Conference Centre, Edinburgh. *Neuro-Oncology* **2018**, *20*, p. i1.

[32] R. C. Martin , C. R. Scoggins , M. Schreeder , W. S. Rilling , C. J. Laing , C. M. Tatum , L. R. Kelly , R. D. Garcia-Monaco , V. R. Sharma , T. S. Crocenzi , S. M. Strasberg , Cancer 2015, 121, 3649–3658. 26149602 10.1002/cncr.29534

[33] N. Kaneda , H. Nagata , T. Furuta , T. Yokokura , Cancer Res. 1990, 50, 1715–1720. 2306725

[34] Y. Kawato , M. Aonuma , Y. Hirota , H. Kuga , K. Sato , Cancer Res. 1991, 51, 4187–4191. 1651156

[35] J. Y. Douillard , D. Cunningham , A. D. Roth , M. Navarro , R. D. James , P. Karasek , P. Jandik , T. Iveson , J. Carmichael , M. Alakl , G. Gruia , L. Awad , P. Rougier , Lancet 2000, 355, 1041–1047. 10744089 10.1016/s0140-6736(00)02034-1

[36] J. Clavadetscher , E. Indrigo , S. V. Chankeshwara , A. Lilienkampf , M. Bradley , Angew. Chem. 2017, 129, 6968–6972. 10.1002/anie.201702404 28485835

