# Supplementary material for: Bioorthogonal Uncaging of the Active Metabolite of Irinotecan by Palladium‐Functionalized Microdevices
Source: Chemistry. 2018 Nov 8;24(63):16783–90. doi: 10.1002/chem.201803725 (PMC6282958; doi:10.1002/chem.201803725)
Supplement: Supplementary file 1 — Supplementary [file CHEM-24-16783-s001.pdf]

CHEMISTRY
A European Journal

Supporting Information

Bioorthogonal Uncaging of the Active Metabolite of Irinotecan by Palladium-Functionalized Microdevices

Catherine Adam,[a] Ana M. Pérez-López,[a] Lloyd Hamilton,[b] Belén Rubio-Ruiz,[a] Thomas L. Bray,[a] Dirk Sieger,[a, b] Paul M. Brennan,*[a, c] and Asier Unciti-Broceta*[a]

chem_201803725_sm_miscellaneous_information.pdf

Supporting Information

Bioorthogonal Uncaging of the Active Metabolite of Irinotecan by Palladium-Functionalized Microdevices

Catherine Adam, Ana M. Pérez-López, Lloyd Hamilton, Belén Rubio-Ruiz, Thomas L. Bray, Dirk Sieger, Paul M. Brennan* and Asier Unciti-Broceta*

LIST OF CONTENTS

1. Synthesis and characterization of compounds **2a**, **2b**, **2d**, **4**, and **5-9** S3

2. NMR spectra of **2a-d** S6

3. Synthesis and characterization of Pd-microdevices S10

4. Non-biological conversion studies in vitro S10

5. Cell culture S11

   Cytotoxicity dose-response curves S11

   Prodrug-into-drug conversion studies – monotherapy activation S11

   Prodrug-into-drug conversion studies – dual therapy activation S11

   Prodrug-into-drug conversion studies – delayed dual therapy activation S12

   Table S1: Calculated EC_{50} ±SD values for all cell lines / nM S12

6. Supplementary Figures S13

7. References S19

1. Synthesis and characterization of compounds 2a, 2b, 2d, 4, and 5-9

Synthesis of 2a. 20 mg scale, DMF, rt, purified 3% MeOH/CH_2Cl_2 to yield an off-white solid (14 mg, 64%). ^{1}H NMR (500 MHz, DMSO-d_6) δ 8.11 (d, J = 9.2 Hz, 1H), 7.63 (d, J = 2.8 Hz, 1H), 7.55 (dd, J = 9.2, 2.8 Hz, 1H), 6.51 (s, 1H), 5.43 (d, J = 2.1 Hz, 2H), 5.31 (s, 2H), 5.08 (d, J = 2.4 Hz, 2H), 3.65 (t, J = 2.4 Hz, 1H), 3.20 (q, J = 7.5 Hz, 2H), 1.87 (m, 2H), 1.34 (t, J = 7.5 Hz, 3H), 0.88 (t, J = 7.3 Hz, 3H); ^{13}C NMR (126 MHz, DMSO-d_6) δ 173.0, 157.3, 156.4, 150.6, 150.4, 146.7, 145.1, 144.6, 132.0, 128.9, 128.1, 122.8, 118.8, 104.6, 96.6, 79.3, 79.2, 72.9, 65.7, 56.4, 50.0, 30.7, 22.8, 14.0, 8.2; HRMS (ESI$^+$) *m/z* [M+H]$^+$ calcd for $C_{25}H_{23}N_2O_5$: 431.1601; found: 431.1597. Purity: 100% (UPLC, method 1).

Synthesis of 2b. 28 mg scale, DMF, rt, purified 2.5% MeOH/ CH_2Cl_2 to yield off-white solid (2.5 mg, 5%). ^{1}H NMR (500 MHz, DMSO-d_6) δ 8.09 (d, J = 9.2 Hz, 1H), 7.60 (d, J = 2.7 Hz, 1H), 7.56 (dd, J = 9.2, 2.7 Hz, 1H), 7.53 – 7.48 (m, 2H), 7.27 (s, 1H), 7.06 – 7.01 (m, 2H), 6.48 (s, 1H), 5.42 (d, J = 1.3 Hz, 2H), 5.31 (s, 2H), 5.29 (s, 2H), 4.81 (d, J = 2.4 Hz, 2H), 3.56 (t, J = 2.4 Hz, 1H), 3.19 (q, J = 7.6, 2H), 1.86 (m, 2H), 1.28 (t, J = 7.6 Hz, 3H), 0.88 (t, J = 7.3 Hz, 4H); ^{13}C NMR (126 MHz, DMSO-d_6) δ 173.0, 157.7, 157.5, 157.3, 150.5, 150.1, 146.8, 144.9, 144.4, 131.9, 130.2, 129.8, 128.9, 128.3, 123.2, 118.7, 115.3, 104.2, 96.5, 79.7, 78.7, 72.9, 70.0, 65.7, 55.9, 50.0, 30.7, 22.7, 13.4, 8.2; HRMS (ESI$^+$) *m/z* [M+H]$^+$ calcd for $C_{32}H_{29}N_2O_6$: 537.2020; found: 537.2025. Purity: 97% (UPLC, method 1).

Synthesis of 2d. 20 mg scale, MeCN, μw, purified 4% MeOH/ CH_2Cl_2 to yield a yellow solid (14 mg, 54%). ^{1}H NMR (500 MHz, DMSO-d_6) δ 8.11 (d, J = 9.2 Hz, 1H), 7.71 (d, J = 2.8 Hz, 1H), 7.57 (dd, J = 9.2, 2.8 Hz, 1H), 7.29 (s, 1H), 7.20 (dd, J = 8.1, 7.6 Hz, 1H), 7.12 (d, J = 7.6 Hz, 2H), 6.49 (s, 1H), 5.44 (s, 2H), 5.33 (s, 4H), 3.24 (q, J = 7.6 Hz, 2H), 2.40 (s, 6H), 1.88 (m, 2H), 1.35 (t, J = 7.6 Hz, 3H), 0.89 (t, J = 7.3 Hz, 3H); ^{13}C NMR (126 MHz, DMSO-d_6) *δ* 172.5, 157.8, 156.8, 150.1, 149.6, 146.3, 144.5, 144.0, 137.9, 132.5, 131.5, 128.5, 128.4, 128.1, 127.9, 122.5, 118.2, 103.4, 96.0, 72.4, 65.2, 65.1, 49.5, 30.2, 22.2, 19.3, 13.5, 7.8; HRMS (ESI$^+$) *m/z* [M+H]$^+$ calcd for $C_{31}H_{31}N_2O_5$: 511.2228; found: 511.2208. Purity: 98% (UPLC, method 1).

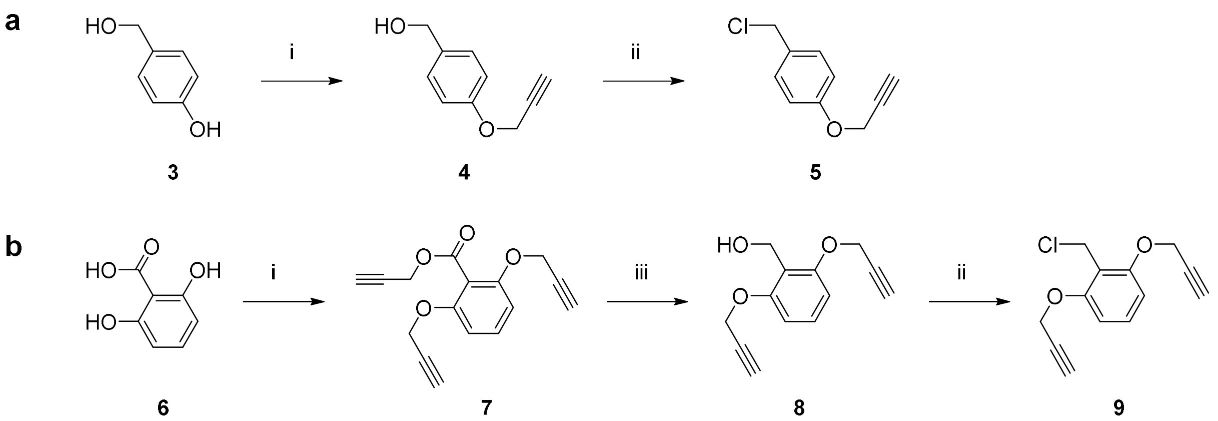

Scheme S1. Synthesis of alkyl halides **5** and **9.** *Reagents and conditions:* (i) Propargyl bromide, K_2CO_3, DMF or acetone, rt or reflux, overnight; (ii) cyanuric chloride, DMF, 1 h, rt, then; benzyl alcohol **4** or **8**, CH_2Cl_2, rt, overnight. (iii) $LiAlH_4$, THF, 0 °C to rt, overnight.

4-Propargyloxybenzyl alcohol (4) was synthesized as previously published[1]

Synthesis of 4-Propargyloxybenzyl chloride (5). Method derived from De Luca et al.[2] Cyanuric chloride (663 mg, 3.67 mmol) was stirred as a suspension in DMF (700 µL) for 1 hr, after which *p*-propargyloxybenzyl alcohol (542 mg, 3.34 mmol) in CH_2CL_2 (5 mL) was added and the reaction stirred at ambient temperature overnight. The reaction was diluted with CH_2CL_2 (25 mL) and washed with sat. bicarb. The organic phase was extracted with CH_2CL_2 (2 ×15 mL), dried over $MgSO_4$ and concentrated *in vacuo*. The crude product was further purified with column chromatography (20-60% EtOAc/*n*-hexane) to yield the title compound as a colourless oil (501 mg, 2.77 mmol, 83%). ^{1}H NMR (500 MHz, $CDCl_3$) δ 7.33 (d, *J* = 8.6 Hz, 2H), 6.96 (d, *J* = 8.7 Hz, 2H), 4.69 (d, *J* = 2.4 Hz, 2H), 4.56 (s, 2H), 2.52 (t, *J* = 2.4 Hz, 1H); ^{13}C NMR (126 MHz, $CDCl_3$) δ 157.7, 130.8, 130.2, 115.3, 78.5, 75.8, 56.0, 46.2.

Synthesis of propargyl 2,6-bis(propargyloxy)benzoate (7). 2,6-Dihydroxybenzoic acid (6.96 g, 45 mmol) and potassium carbonate (30.5 g, 220 mmol) were suspended in dry DMF (40 mL) and stirred for 30 min at 0 °C. Propargyl bromide (21 mL, 80% *v/v* in toluene, 141 mmol) was added dropwise and the reaction was warmed to ambient temperature and stirred for 3 d. The reaction was diluted with water (300 mL) and extracted with diethyl ether (6 × 200 mL). The combined organic phases were washed with brine, dried over anhydrous $MgSO_4$ and concentrated *in vacuo* to yield the title compound as a brown oil (5.37 g, 20.1 mmol, 44%), used without further purification. ^{1}H NMR (400 MHz, $CDCl_3$) δ 7.32 (t, *J* = 8.4 Hz, 1H), 6.76 (d, *J* = 8.4 Hz, 2H), 4.91 (d, *J* = 2.5 Hz, 2H), 4.71 (d, *J* = 2.5 Hz, 4H), 2.51 (t, *J* = 2.4 Hz, 2H), 2.50 (t, *J* = 2.5 Hz, 1H); ^{13}C NMR (126 MHz, $CDCl_3$) δ 165.1, 155.9, 131.3, 114.0, 106.8, 78.2, 77.7, 76.2, 75.2, 57.0, 52.9.

Synthesis of 2,6-bis(propargyloxy)benzyl alcohol (8). Intermediate **7** (5.37 g, 20 mmol) was dissolved in THF and cooled to 0 ºC for the addition of $LiAlH_4$ (1 M in THF, 24 mL) before warming to ambient temperature and stirring overnight. The reaction was quenched at 0 ºC with 10% NaOH (40 mL), stirring for 30 min. The aqueous phase was extracted with CH_2Cl_2 (3 × 70 mL) and the combined organic phases washed with brine (40 mL), dried over anhydrous $MgSO_4$ and concentrated *in vacuo*. The crude alcohol was purified by flash column chromatography (30% EtOAc/*n*-hexane) to yield the title compound as a white solid (2.74 g, 12.6 mmol, 63%). ^{1}H NMR (500 MHz, $CDCl_3$) δ 7.24 (t, *J* = 8.4 Hz, 1H), 6.72 (d, *J* = 8.4 Hz, 2H), 4.81 (d, *J* = 6.7 Hz, 2H), 4.74 (d, *J* = 2.4 Hz, 4H), 2.51 (t, *J* = 2.4 Hz, 2H), 2.37 (t, *J* = 6.7 Hz, 1H); ^{13}C NMR (126 MHz, $CDCl_3$) δ 162.5, 155.7, 129.1, 106.6, 78.5, 75.8, 56.6, 54.4; HRMS (ESI$^+$) m/z $[M+H]^+$ calcd for $C_{13}H_{13}O_3$: 217.0865, found: 217.0859.

Synthesis of 2,6-bis(propargyloxy)benzyl chloride (9). Cyanuric chloride (180 mg, 1.00 mmol) was stirred as a suspension in DMF (0.1 mL) for 1 h. Intermediate **8** (194 mg, 0.90 mmol) in CH_2Cl_2 (1 mL) was added and the reaction stirred at ambient temperature overnight. The reaction was diluted with CH_2Cl_2 (25 mL) and washed with a saturated $NaHCO_3$ solution. The organic phase was extracted with CH_2Cl_2 (2 ×15 mL), dried over anhydrous $MgSO_4$ and concentrated *in vacuo*. The crude product was purified with column chromatography (40% EtOAc/*n*-hexane) to yield the title compound as a white solid (161 mg, 77%). ^{1}H NMR (500 MHz, $CDCl_3$) 7.28 (t, *J* = 8.4 Hz, 1H), 6.72 (d, *J* = 8.4 Hz, 2H), 4.78 (d, *J* = 2.3 Hz, 4H), 4.78 (s, 2H), 2.51 (t, *J* = 2.4 Hz, 2H); ^{13}C NMR (126 MHz, $CDCl_3$) δ 156.7, 123.0, 115.9, 106.2, 78.4, 75.7, 56.6, 35.3.

2. NMR spectra of 2a-d

^{1}H NMR spectra of **2a** in DMSO-d_6 at 300 K

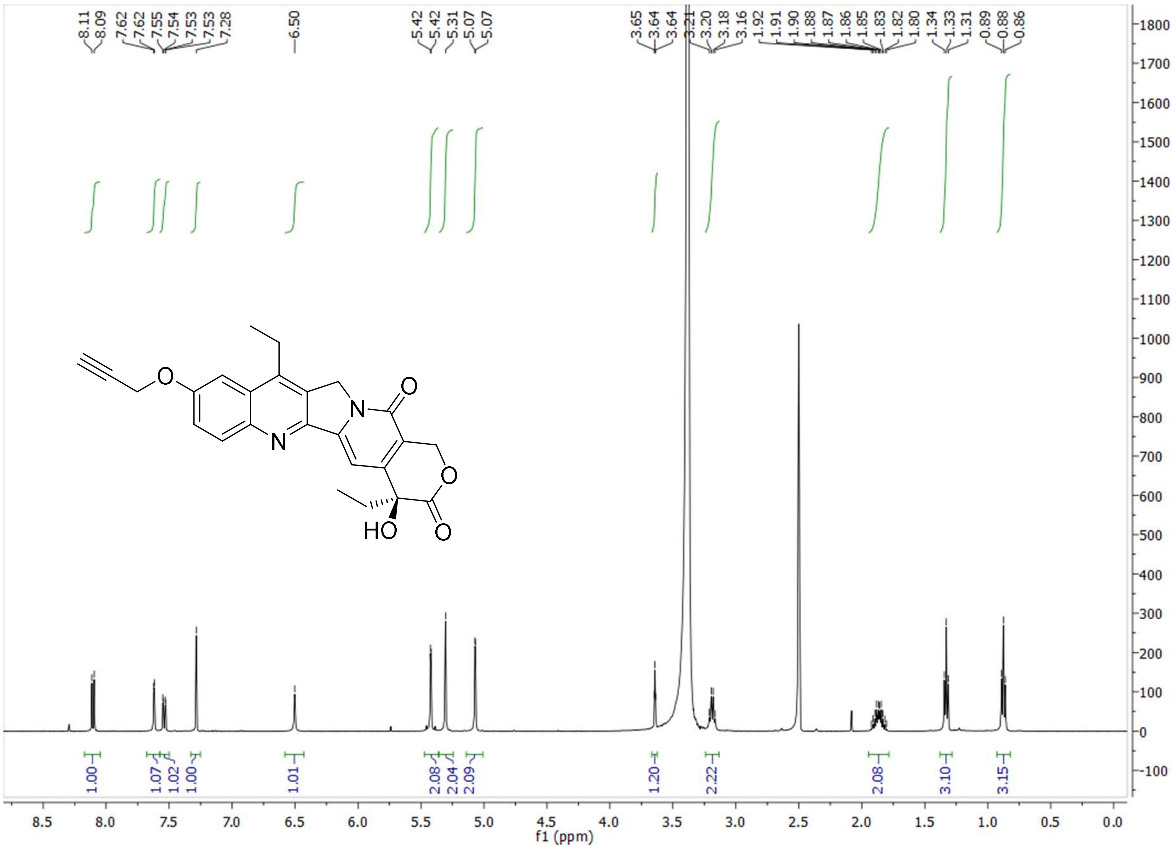

^{13}C NMR spectra of **2a** in DMSO-d_6 at 300 K

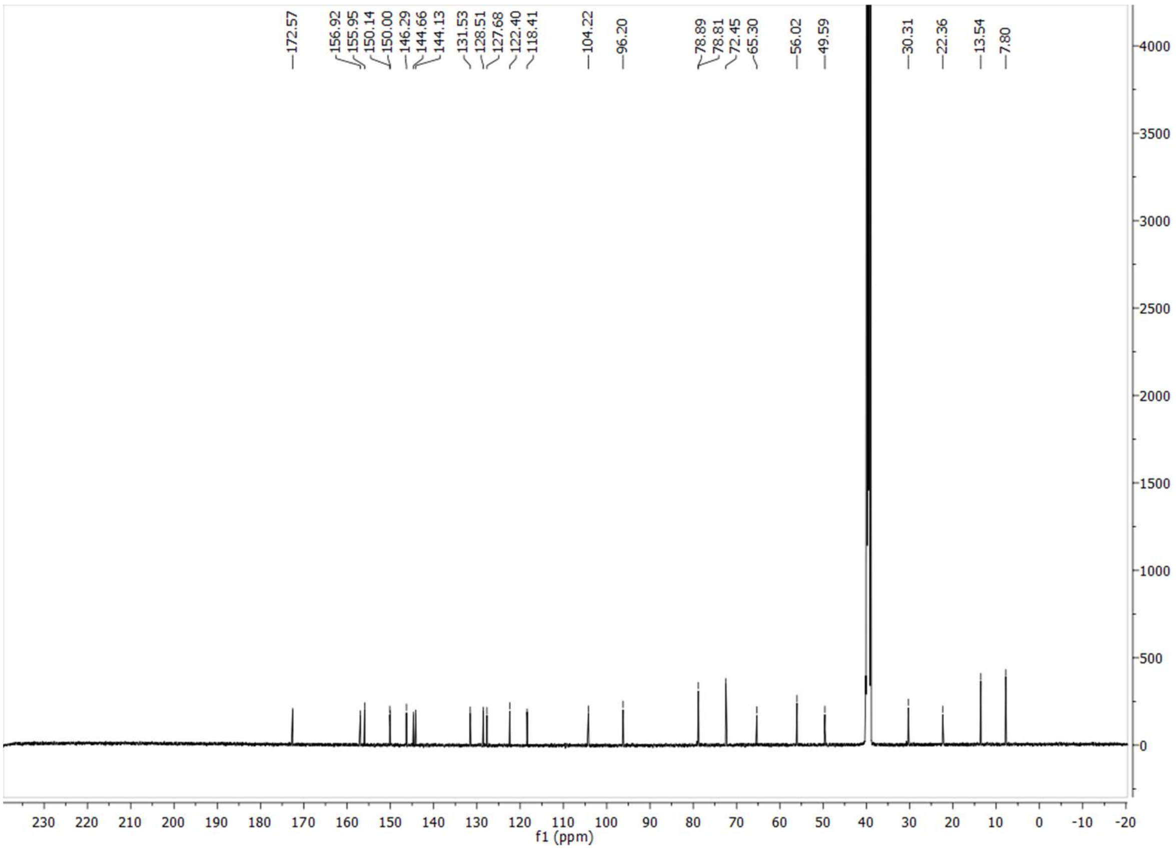

^{1}H NMR spectra of **2b** in DMSO-d_6 at 300 K

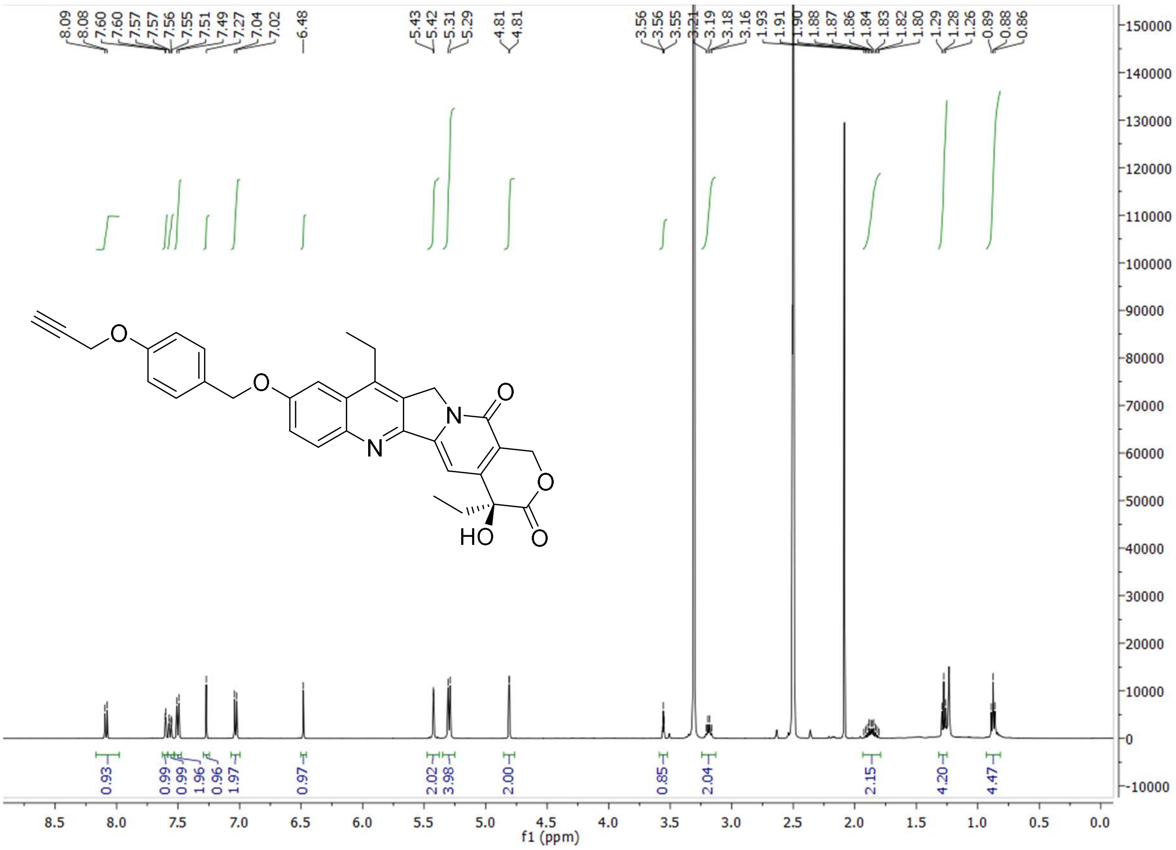

^{13}C NMR spectra of **2b** in DMSO-d_6 at 300 K

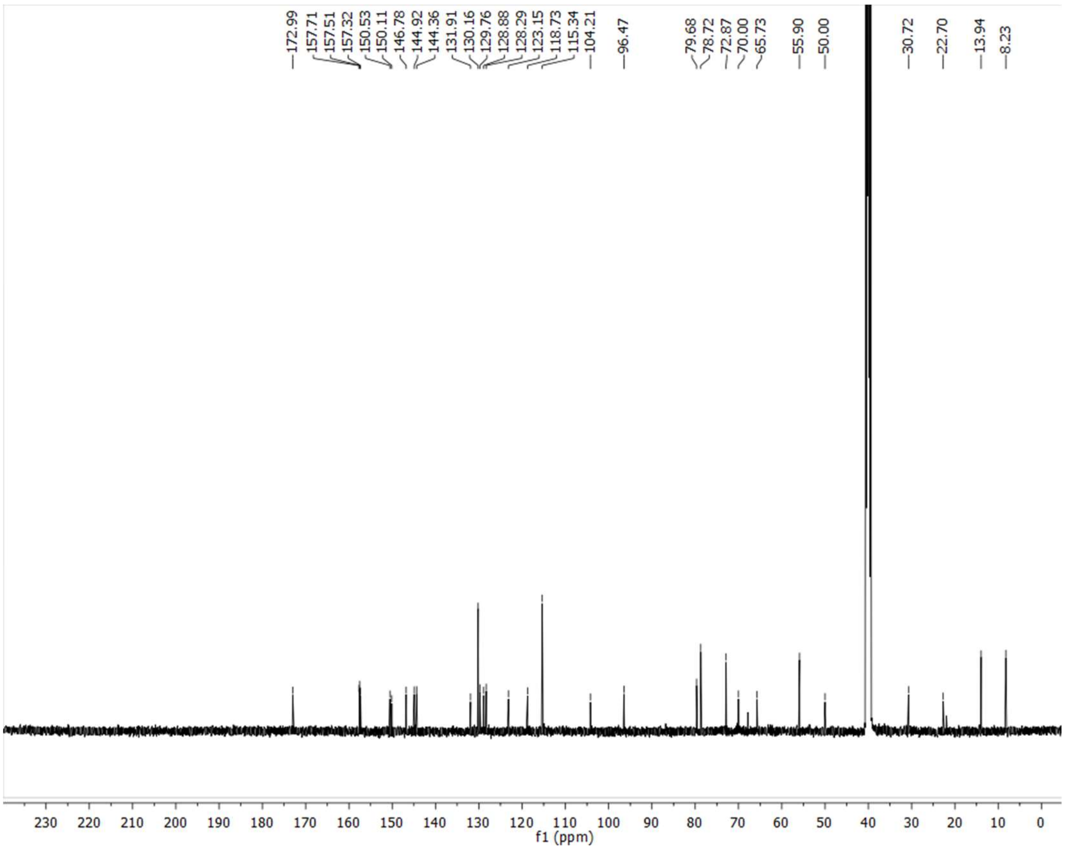

^{1}H NMR spectra of **2c** in DMSO-d_6 at 300 K

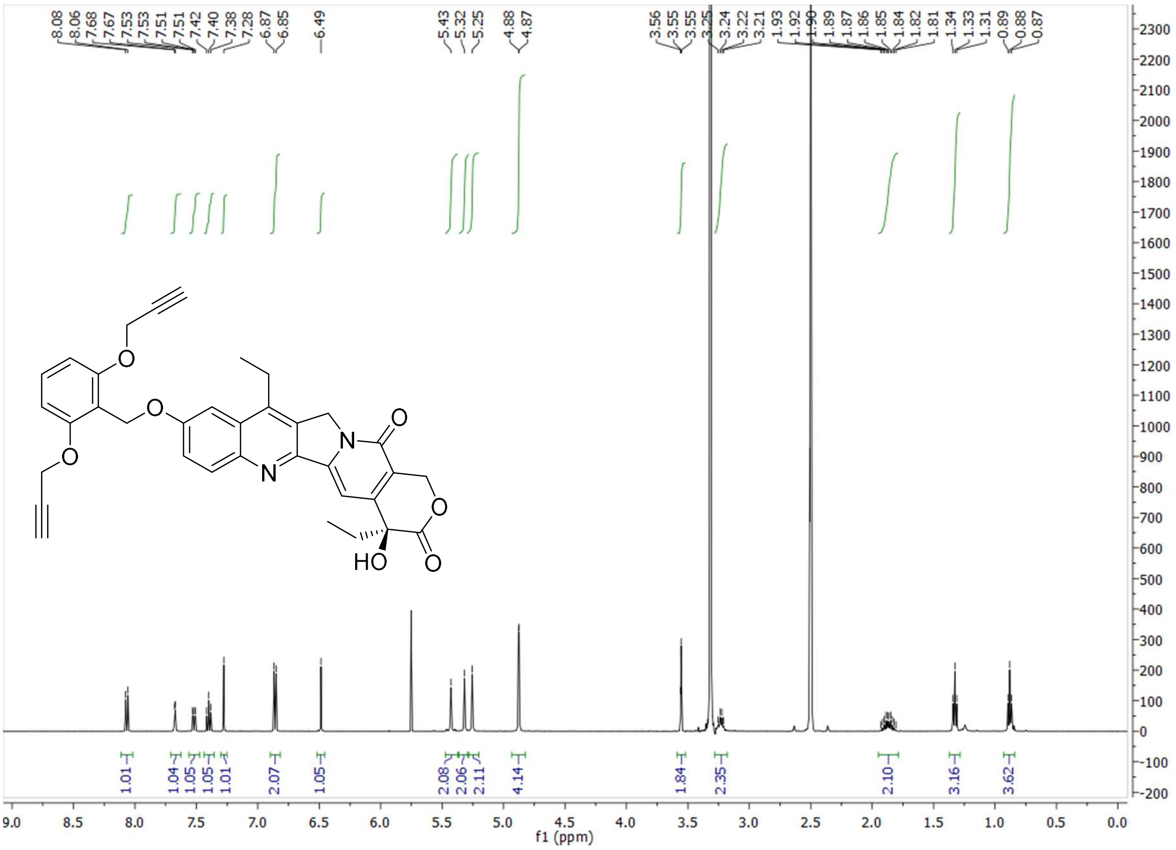

^{13}C NMR spectra of **2c** in DMSO-d_6 at 300 K

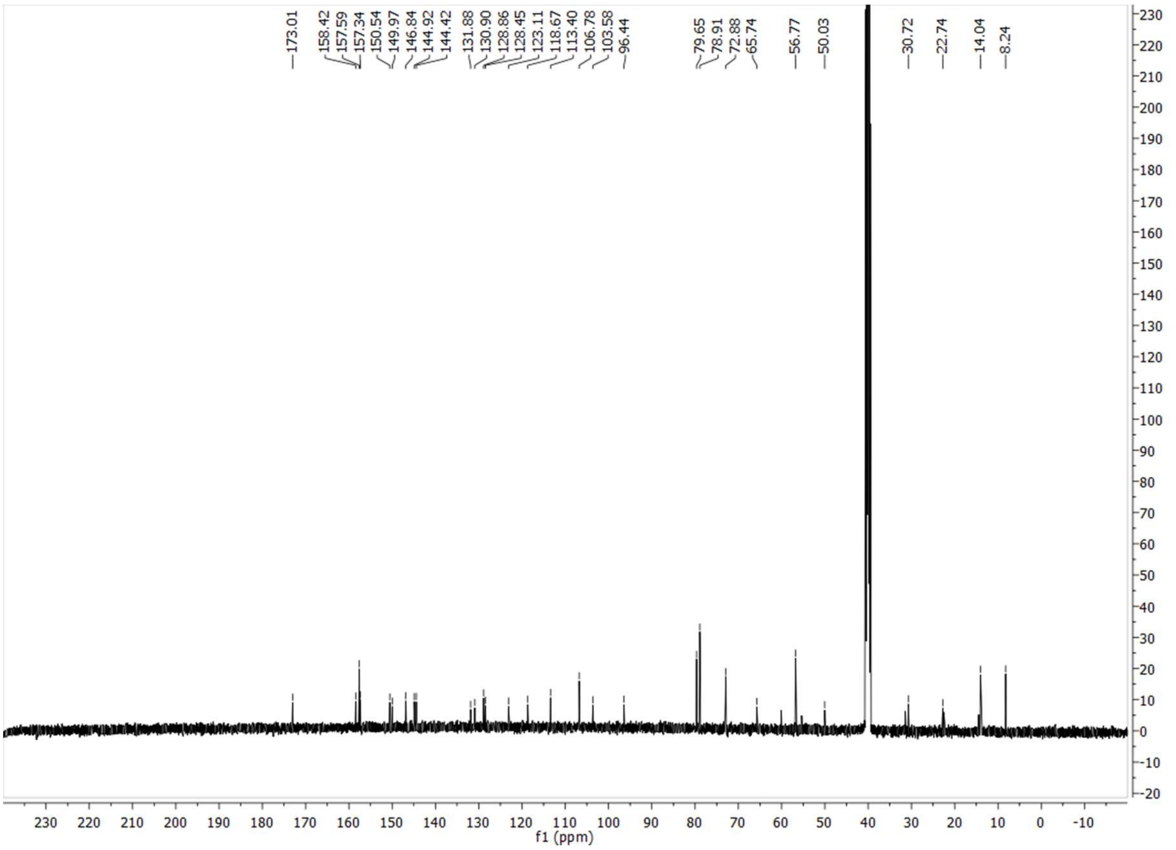

^{1}H NMR spectra of **2d** in DMSO-d$_6$ at 300 K

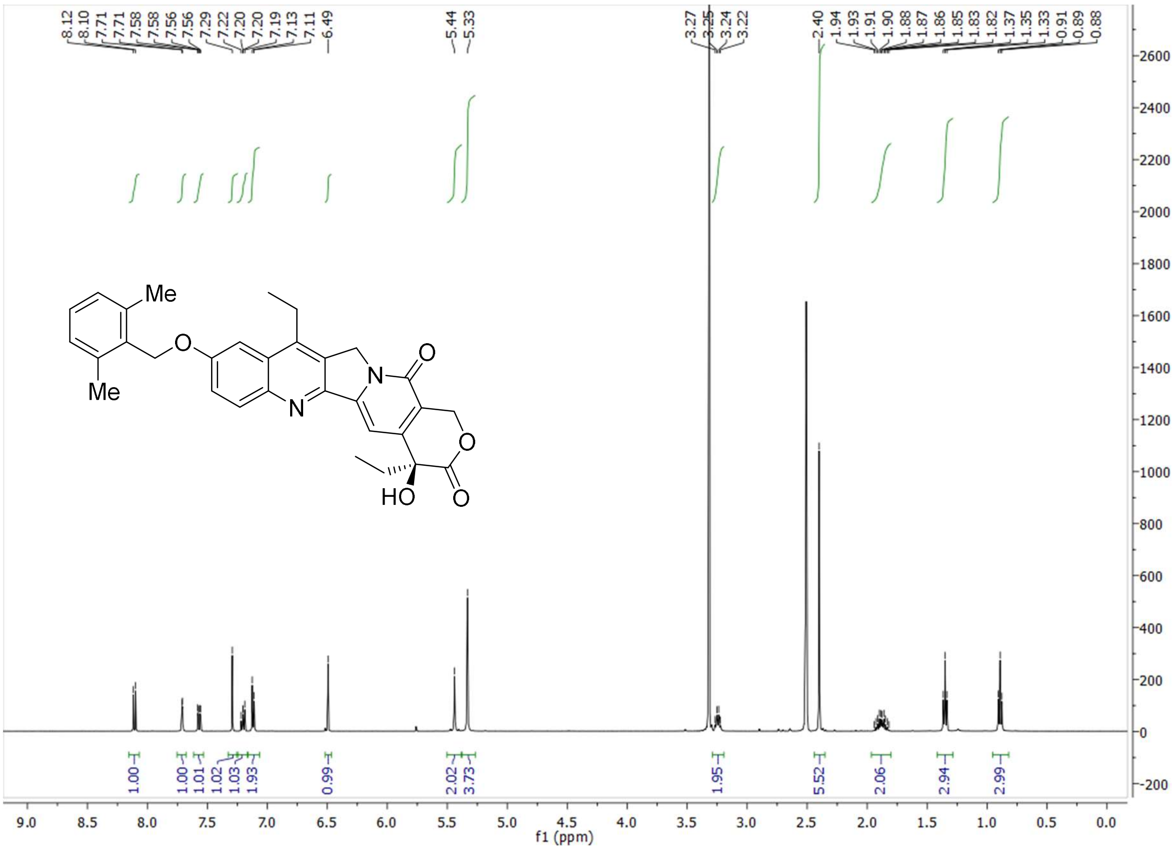

^{13}C NMR spectra of **2d** in DMSO-d$_6$ at 300 K

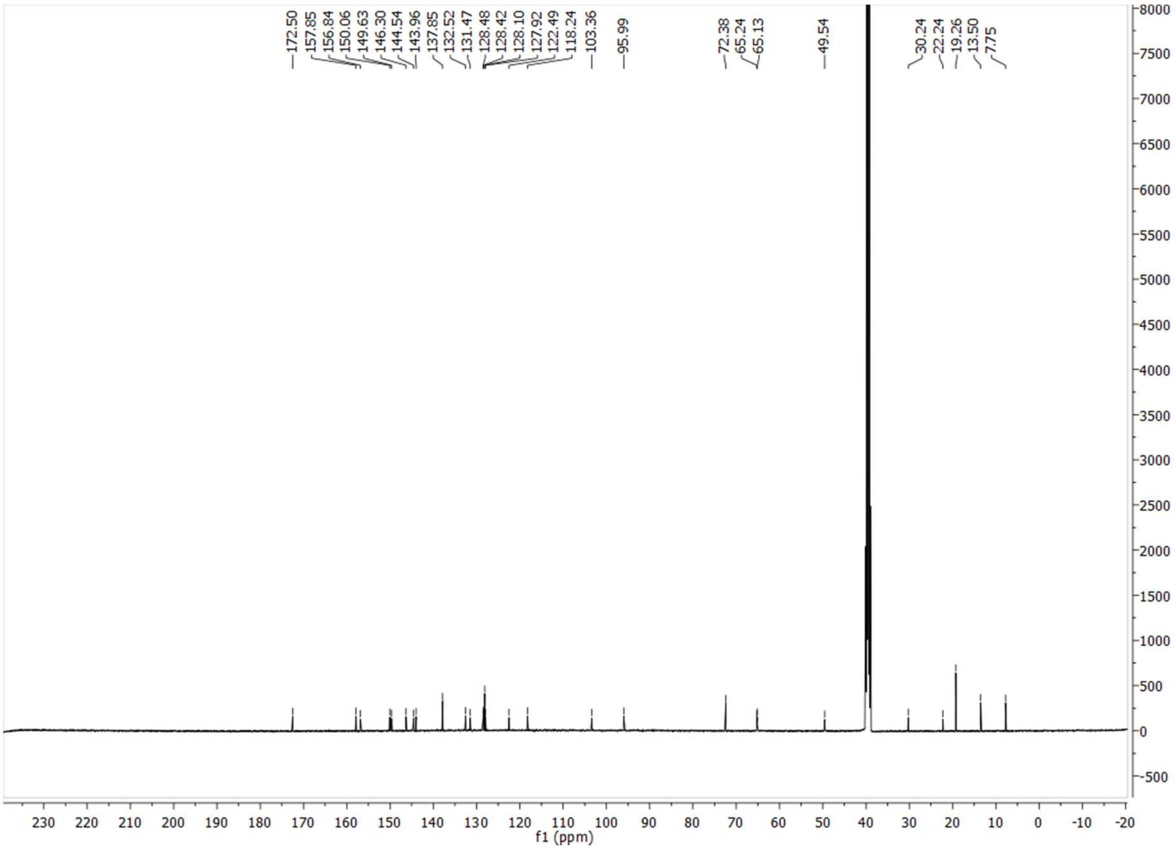

3. Synthesis and characterization of Pd-microdevices

NovaSyn TG amino resins HL (1.6 g, 0.24 NH_2 mmol/g, particle size 30 µm) and palladium acetate (263 mg, 1.17 mmol) were added into a 25 mL Biotage microwave vial (Biotage). Next, toluene (10 mL) was added to the vial and heated to 80 °C for 10 min, then stirred at room temperature for an additional 2 h. The resins were filtered and washed with CH_2Cl_2 (5 x 20 mL) and MeOH (5 x 20 mL). Resins were dispersed in 10 % hydrazine monohydrate in MeOH (10 mL) and stirred at room temperature for 25 min. The resins were again filtered and washed with MeOH (5 x 20 mL) and CH_2Cl_2 (5x 20 mL). Resins were added to a solution of Fmoc-Glu(OH)-OH (216 mg, 0.59 mmol), Oxyma (166 mg, 1.17 mmol), *N*,*N*'-diisopropylcarbodiimide (DIC) (143 µL, 1.17 mmol) with CH_2Cl_2/DMF (2:1, 9 mL) and stirred for 2 h at rt. The resins were filtered and washed with CH_2Cl_2 (5 x 20 mL), MeOH (5 x 20 mL) and water (5 x 20 mL). Finally, resins were dispersed and shaken in a solution of acetic anhydride (60 µL) in DCM (10 mL) for 1 h at rt. The solvents were filtered and the resins were washed with CH_2Cl_2 (3 × 10 mL) and methanol (3 × 10 mL) and dried under vacuum at 40 °C for 3 d. Completed coupling was verified by ninhydrin test after the final MeOH wash.

4. Non-biological conversion studies in vitro

Stock solutions of **2c**, **2d** (-ve control) and **1** (+ve control) at 100 µM in 10% *v/v* serum/PBS (1000-fold dilution from 100 mM DMSO stock solution) were added to Eppendorf microcentrifuge tubes containing: a) no resins (control experiment); b) 1 mg/mL of 30 µm NovaSyn TG amino resins HL (aka naked beads, control experiment); and c) 1 mg/mL of Pd-devices as synthesized in section **3**. Vials were incubated 37 ºC and shaken at 1200 rpm. **Fluorescence Analysis:** 3 × 2 µL aliquots of each reaction solution were analysed at regular intervals. Measurements were recorded on a *Thermo Scientific™ NanoDrop 2000 UV-Vis spectrophotometer* using an excitation wavelength of 365 nm and recording emission between 395-751 nm wavelengths. The data (average of three measurements for each data point) was fit to a one phase decay equation: **Y=(Y0 −Plateau) × exp(-K×X) + Plateau**, setting Y0 to 0, using *GraphPad Prism 5*. **TLC Analysis:** 250 µL aliquots of each reaction solution were taken after 1 d, centrifuged to separate the resins, and the supernatant removed and dried (freeze dryer, overnight). The resulting solid was re-suspended in 5% MeOH / CH_2Cl_2 (60 µL), sonicated and centrifuged. The TLC of the supernatant (5% MeOH / CH_2Cl_2) was visualized with a 254 nm UV lamp. **HPLC Analysis:** 250 µL aliquots of each reaction solution were taken after 1 d, centrifuged to separate the devices, and the supernatant removed and dried (freeze dryer, overnight). The resulting solid was re-suspended in 250 µL MeOH, centrifuged and analysed. **Column conditions:** Retention times were recorded by analytical reverse phase HPLC analysis using a Waters 600E (100 µL) gradient pump using a 717plus

autosampler and a Waters 996 PDA (210 – 400 nm) equipped with a Phenomenex Luna C18(2), 5 µm, 250 x 4.6 mm column at a flow rate of 1 mL / min with an injection volume of 10 µL. **Gradient elution:**

Time (min)	% H_2O + 0.1% TFA	% MeCN + 0.1% TFA
0	95	5
30	5	95
35	5	95
40	95	5
50	95	5

5. Cell culture

Cell lines were grown in culture media supplemented with 10% Fetal Bovine Serum (FBS) and 2 mM L-glutamine, incubated in a tissue culture incubator at 37 °C and 5% CO_2. Human colorectal cancer (HCT116) were cultured in McCoy's 5A medium and human glioblastoma multiforme U-87 (gifted from Dr Noor Gamon) and U-251 cells were cultured in Dulbecco's modified Eagle's media (DMEM).

Cytotoxicity dose-response curves

Cells were seeded in a 96 well plate format at 1500 (HCT116) or 2000 (U-87 and U-251) cells / well and incubated for 48 h before treatment. Each well was then replaced with fresh media (95 µL) followed by the compound of interest (5 µL in 2% *v/v* DMSO/ medium at 20× the final concentration). Control wells incubated with DMSO (0.1 % *v*/*v*). Cells were then incubated 5 d. Cell viability was measured on day 5 by adding PrestoBlue® cell viability reagent (10 µL) to each well and incubating for 90 min. Fluorescence emission was detected using a PerkinElmer EnVision 2101 multilabel reader (Ex / Em: 540 nm / 590 nm). All conditions were normalized to control wells (100%) and curves fitted with *GraphPad Prism 5* using a sigmoidal variable slope curve. Plates were repeated in triplicate for each compound.

Prodrug-into-drug conversion studies – monotherapy activation

Cells were plated as described and incubated for 48 h prior to treatment. Each well was then replaced with fresh media (95 µL) or fresh media containing Pd-devices (95 µL plus 0.1 mg devices) for prodrug activation wells. The compound of interest (5 µL in 2% *v/v* DMSO/ medium at 20× the final concentration). Control wells incubated with DMSO (0.1 % *v*/*v*), with 1 mg/mL Pd-resins for Pd control wells. All cells were incubated for 5 d and cell viability was measured as before. Plates were repeated in triplicate for each experiment.

Prodrug-into-drug conversion studies – dual therapy activation

Cells were plated as described and incubated for 48 h prior to treatment. Each well was then replaced with fresh media (90 µL) or fresh media containing Pd-devices (90 µL plus 0.1 mg devices) for pro-drug

activation wells. **5FU** / **Pro-5FU** were added to relevant wells (5 µL in 2% *v/v* DMSO / medium at 20× the final concentration) followed by **1** / **2c** for relevant wells (5 µL in 2% *v/v* DMSO / medium at 20× the final concentration). Control wells with only one drug / prodrug were topped up to the correct volume (5 µL of 2% *v/v* DMSO / medium). Control wells incubated with DMSO (0.2 % *v*/*v*), with 1 mg/mL Pd-devices for Pd control wells. All cells were incubated for 5 d and cell viability was measured as before. To ensure appropriate controls, each combination activation assay plate had the following wells in triplicate: DMSO (0.1% *v/v*); Pd-devices (1 mg/mL); Pro-5FU alone (100 µM); **2c** alone (10 or 100 nM for HCT116 and U-87 respectively); 5FU alone (30 and 100 µM); **1** alone (10 or 100 nM for HCT116 and U-87 respectively); Pro-5FU+**2c** combination (100 µM Pro-5FU + 10 or 100 nM **2c** for HCT116 and U-87 respectively); 5FU+**1** (30 and 100 µM 5FU + 10 or 100 nM **1** for HCT116 and U-87 respectively); and Pro-5FU+**2c**+Pd-devices combination activation assay (30 and 100 µM Pro-5FU + 10 or 100 nM **2c** for HCT116 and U-87 respectively). Plates were repeated in triplicate for each experiment.

Prodrug-into-drug conversion studies – delayed dual therapy activation

Cells were plated as described above and incubated for 48 h prior to treatment. Each well was then replaced with fresh media (90 µL) or fresh media containing Pd-devices (90 µL + 0.1 mg devices) for prodrug activation wells. Cells were then treated with both drugs (**1** + 5FU) or both prodrugs (**2c** + Pro-5FU) on day 1, **or** two days apart (*i.e.* one treatment of either **1**/**2c** **or** 5FU/Pro-5FU on day 1 and the second treatment on day 3). Concentrations of treatments remained constant at: [**1**] and [**2c**] = 300 nM, [5FU] and [Pro-5FU] = 100 µM. Control wells with plus 0.2% *v/v* DMSO were also treated simultaneously or two days apart. Cell viability was measured on day 5 as before.

Table S1: Calculated EC_{50} ±SD values for all cell lines / nM

	HCT116	$EC_{50}/EC_{50\,\mathbf{1}}$	U-87	$EC_{50}/EC_{50\,\mathbf{1}}$	U-251	$EC_{50}/EC_{50\,\mathbf{1}}$
Topotecan	75 ±8.6	39	125 ±67	1.3	21 ±3.6	4
Irinotecan	1855 ±650	957	7637 ±3005	81	1672 ±350	286
SN-38 (**1**)	1.9 ±0.35	1	94 ±71	1	5.8 ±1.3	1
**2a**	5.1 ±2.5	3	63 ±45	1	12 ±1.9	2
**2b**	16 ±8.8	9	123 ±51	1	22 ±7.6	4
**2c**	84 ±13	43	1466 ±1052	16	205 ±6.1	35
**2d**	104 ±32	54	603 ±420	6	92 ±11	16
5FU (µM)	2.4 ±0.61	-	20 ±34	-	28 ±6.1	-
pro5FU (µM)	>100	-	>100	-	>100	-
Co-treatment **2c** + pro5FU (µM)	>100	-	>100	-	>100	-

6. Supplementary Figures

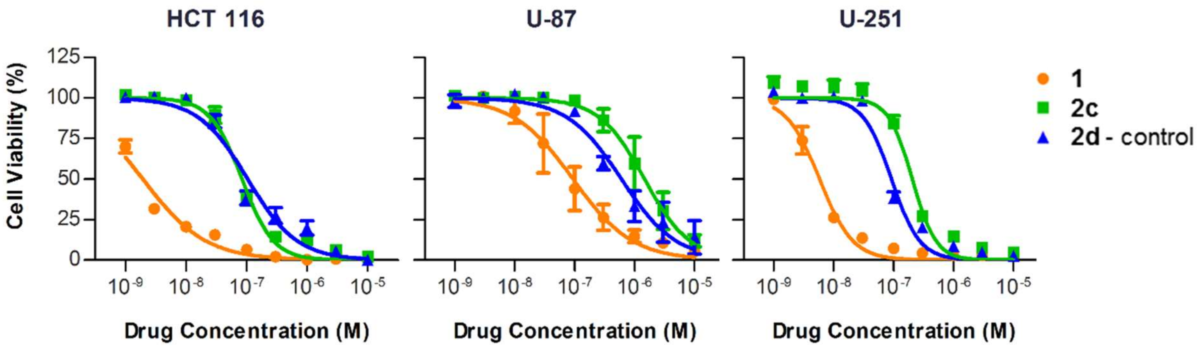

Figure S1. Half-log dose response curves for **1** compared to prodrugs **2c** and **2d** (control) after a 5 d incubation with HCT116, U-87 and U-251 cells. Error bars: ± SEM, n = 3.

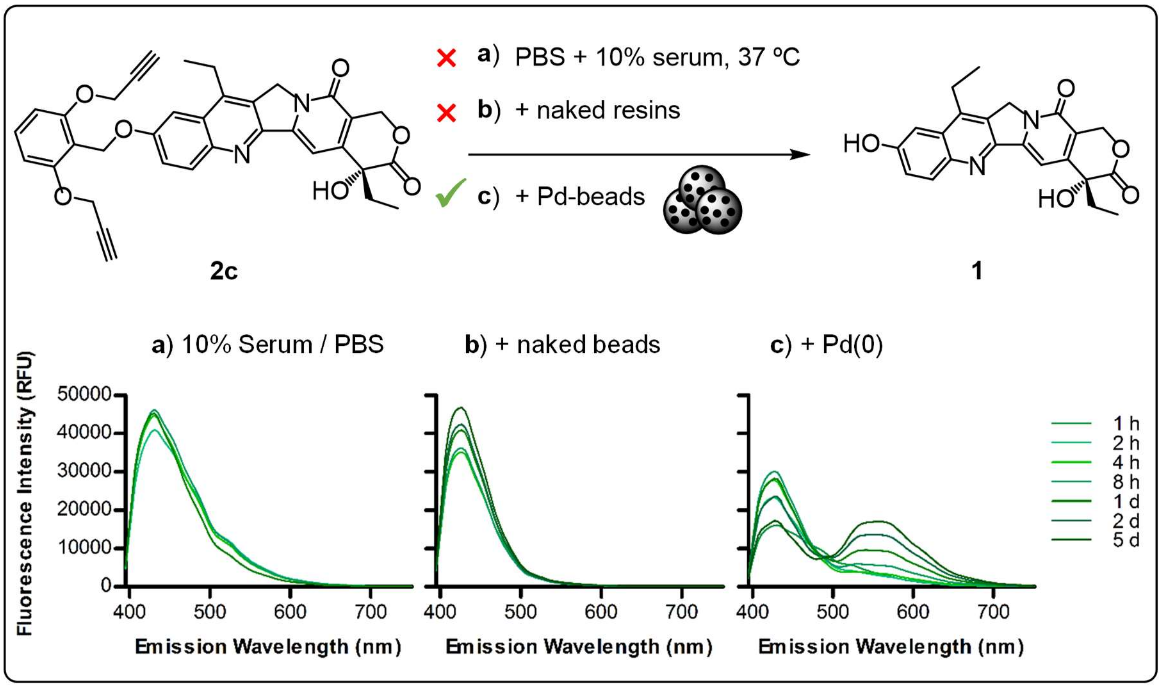

Figure S2. *Control experiments:* (**a**) Fluorescence monitoring of **2c** without beads under biological conditions (100 µM in PBS + 10% serum, 37 ºC). (**b**) Fluorescence monitoring of **2c** with naked beads under biological conditions (100 µM in PBS + 10% serum, 37 ºC, 1 mg/mL naked beads). *Activation assay:* **c)** Fluorescence monitoring of **2c** with Pd-devices under biological conditions (100 µM in PBS + 10% serum, 37 ºC, 1 mg/mL Pd-devices).

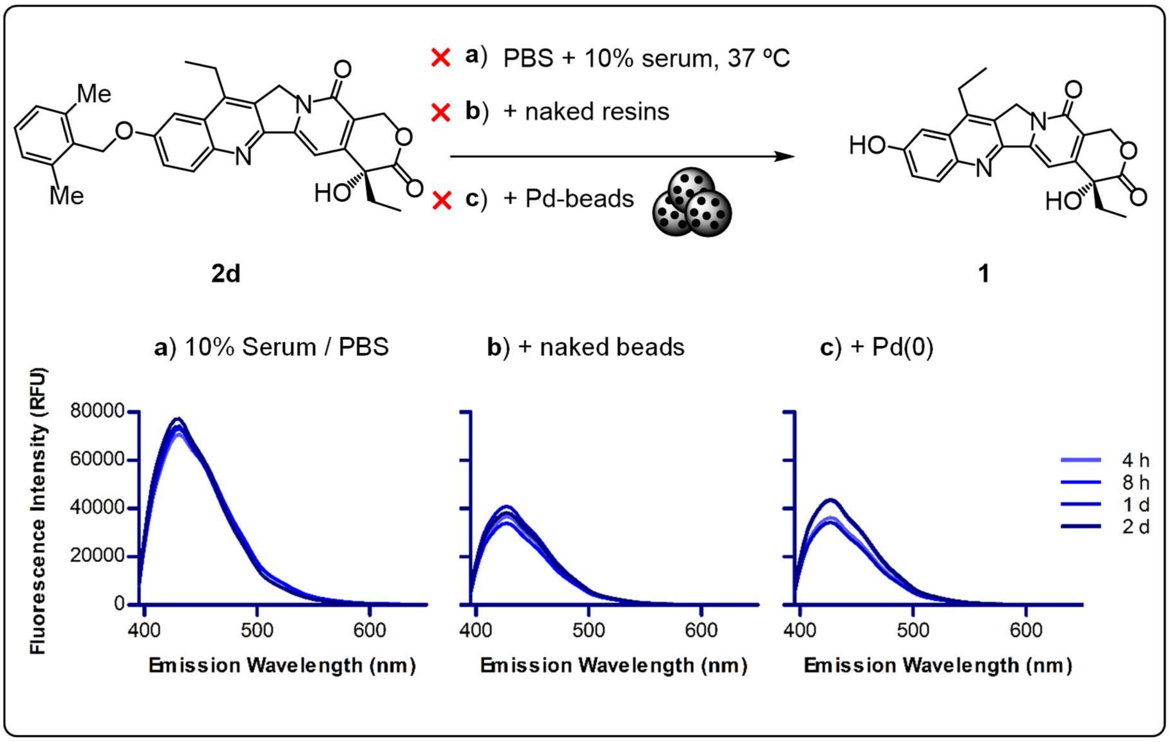

Figure S3. *Control experiments*: (**a**) Fluorescence monitoring of **2d** without beads under biological conditions (100 µM in PBS + 10% serum, 37 ºC). (**b**) Fluorescence monitoring of **2d** with naked beads under biological conditions (100 µM in PBS + 10% serum, 37 ºC, 1 mg/mL naked beads). (**c**) Fluorescence monitoring of **2d** with Pd-devices under biological conditions (100 µM in PBS + 10% serum, 37 ºC, 1 mg/mL Pd-devices) .

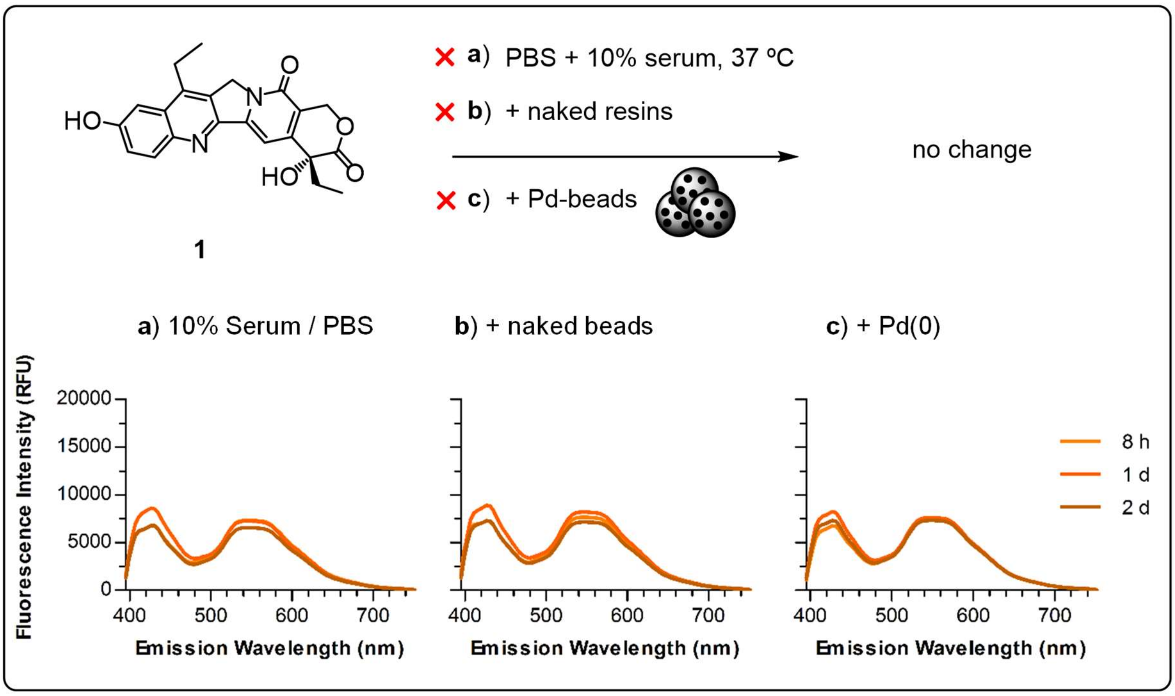

Figure S4. *Control experiments:* (**a**) Fluorescence monitoring of **1** without beads under biological conditions (100 µM in PBS + 10% serum, 37 ºC). (**b**) Fluorescence monitoring of **1** with naked beads under biological conditions (100 µM in PBS + 10% serum, 37 ºC, 1 mg/mL naked beads). (**c**) Fluorescence monitoring of **1** with Pd-devices under biological conditions (100 µM in PBS + 10% serum, 37 ºC, 1 mg/mL Pd-devices).

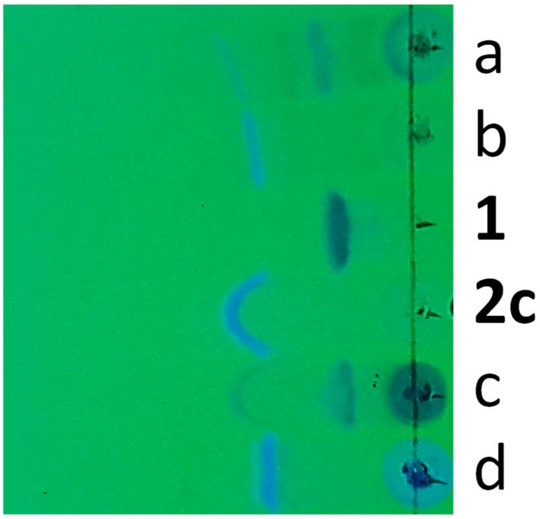

Figure S5. TLC analysis of reaction mixture **2c** + Pd-devices after 1 d at 37 ºC - referenced to **1** and **2c** (middle). Conditions: **a**) 100 µM **2c** in PBS + 1 mg/mL Pd-devices. **b**) *Control Experiment (no beads)* 100 µM **2c** in PBS. **c**) 100 µM **2c** in 10% *v/v* serum / PBS + 1 mg/mL Pd-devices. **d**) *Control Experiment (no beads)* 100 µM **2c** in 10% *v/v* serum / PBS.

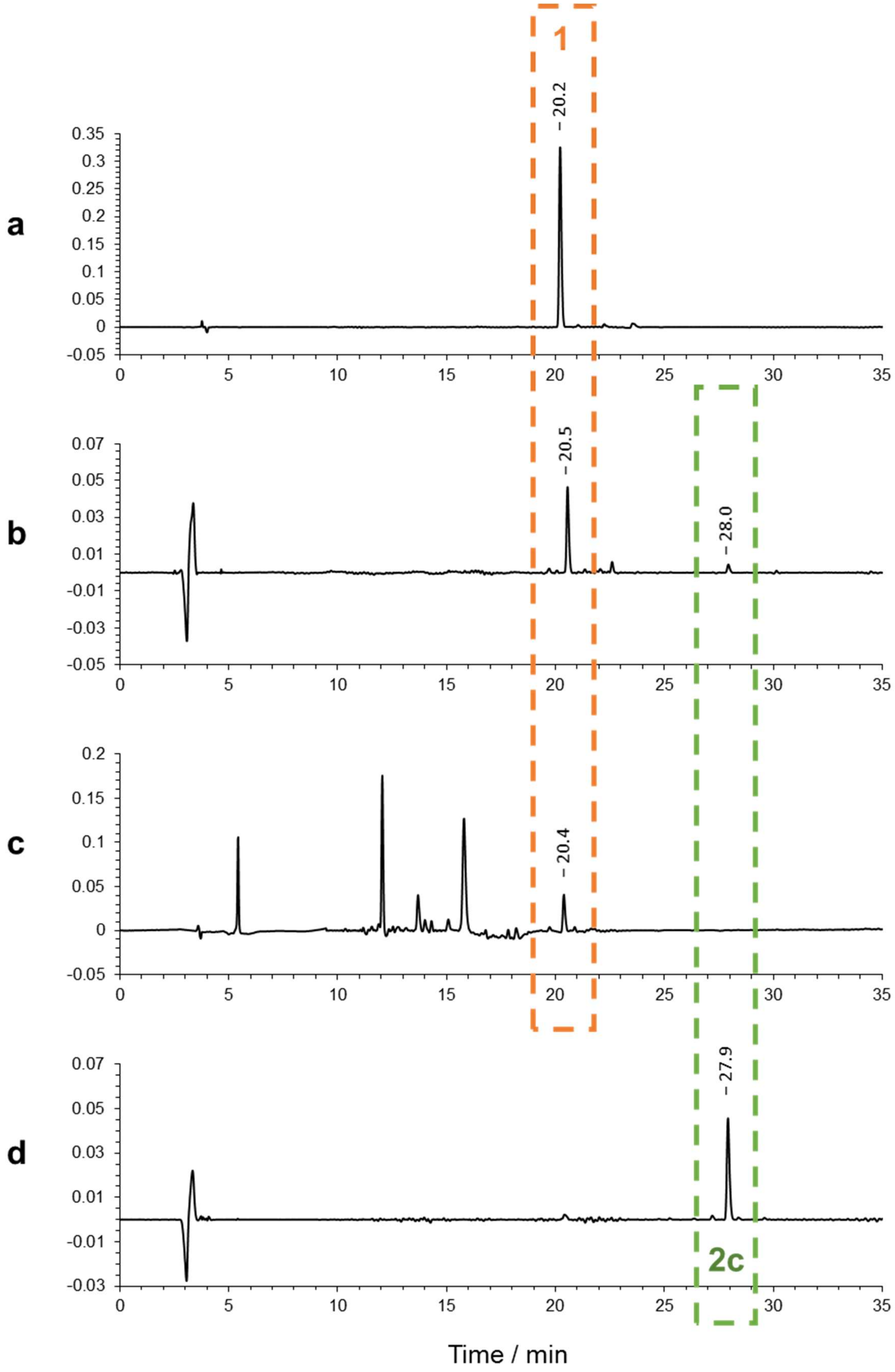

Figure S6. HPLC traces (UV detector 220 nm) of: **a**) Compound **1,** 300 µM in MeOH. **b**) Reaction mixture of **2c** +1 mg/mL Pd-devices in PBS, 37 ºC, 24 h, 1200 rpm. **c**) Reaction mixture of **2c** + 1 mg/mL Pd-devices in PBS/10% serum, 37 ºC, 24 h, 1200 rpm. **d**) **2c** Compound **2c,** 200 µM in MeOH.

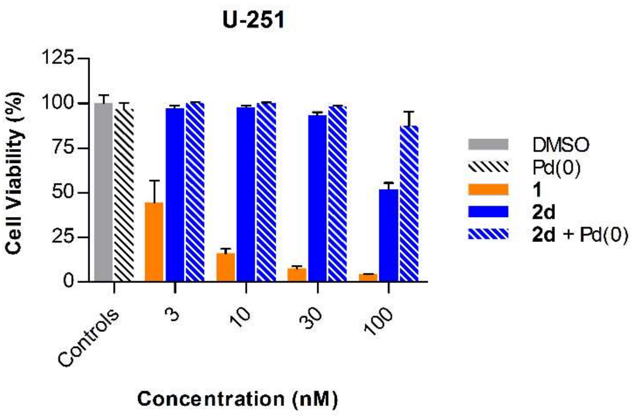

Figure S7. *Control experiment:* Pd-devices + **2d** at nM concentrations in U-251 cells. Experiments: 0.1% (*v/v*) DMSO (control); 1 mg/mL of Pd-devices (–ve control); **1** (+ve control); **2d** (–ve control); 1 mg/mL of Pd-devices + **2d** (activation control assay). Cell viability was measured at day 5 using PrestoBlue reagent. Error bars: ± SEM, n = 3.

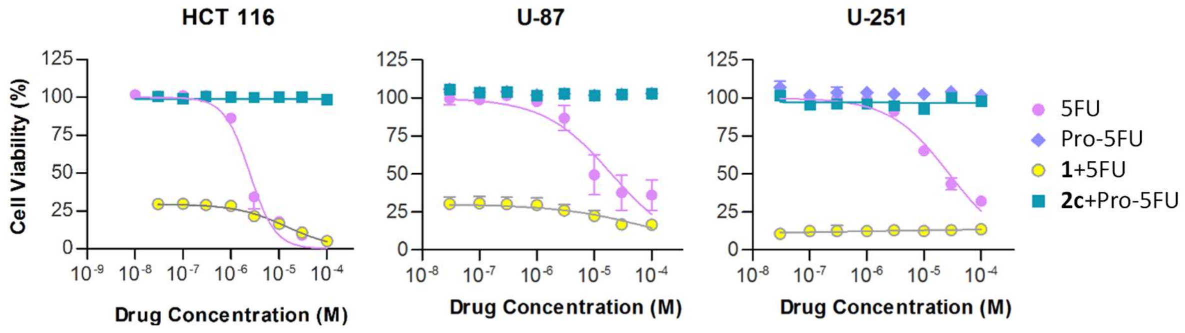

Figure S8. Dose-response curves for 5FU, Pro-5FU, **1**+5FU combination and **2c**+Pro-5FU combination. Concentration of **1** / **2c** remained constant at 10 nM for HCT116, 100 nM for U-87 and 30 nM for U-251 as the concentration of 5FU / pro-5FU was varied. See **Table S1** for calculated EC_{50}. For dose-response curve fitting of **1** + **5FU** combination, sigmoidal dose-response (variable slope) was used, constraining HCT116 to 29.4%; U-87 to 30.1%; and U-251 to 11.9% as the top responses for each cell line. Error bars: ± SEM, n = 3.

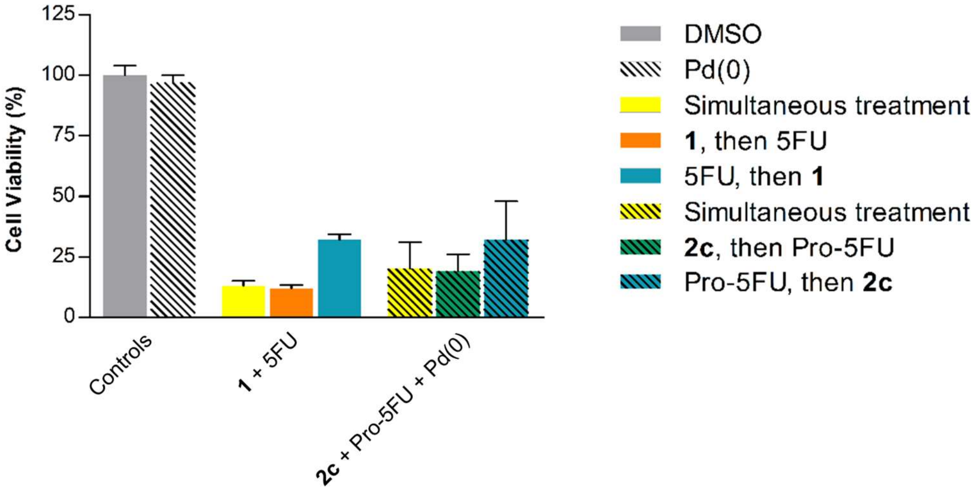

Figure S9. Simultaneous *vs* sequential co-activation of **2c** and Pro-5FU in U-87 cancer cell culture with Pd-microdevices. The day of treating cells with drug / prodrug were varied, while concentrations remained constant at: [**1**] and [**2c**] = 300 nM, [5FU and [Pro-5FU] = 100 μM. Controls: 0.1% (v/v) DMSO (control, grey); 1 mg/mL of Pd-devices (–ve control, black stripes); **1** + 5FU (both added on day 1, yellow); **1** (added on day 1) + 5FU (added on day 3, orange); 5FU (added on day 1, blue) + **1** (added on day 3). Experiments: 1 mg/mL of Pd-devices + **2c** + Pro-5FU (both added on day 1, yellow with black stripes); 1 mg/mL of Pd-devices +**2c** (added on day 1) + Pro-5FU (added on day 3, green with black stripes); 1 mg/mL of Pd-devices + Pro-5FU (added on day 1, blue with black stripes) + **2c** (added on day 3). Error bars: ± SEM.

7. References

[1] B. Rubio-Ruiz, J. T. Weiss, A. Unciti-Broceta, *J. Med. Chem.* **2016**, *59*, 9974-9980.

[2] L. De Luca, G. Giacomelli, A. Porcheddu, *Org. Lett*. **2002**, *4*, 553-555.
